# circVAMP3 Drives CAPRIN1 Phase Separation and Inhibits Hepatocellular Carcinoma by Suppressing c‐Myc Translation

**DOI:** 10.1002/advs.202103817

**Published:** 2022-01-24

**Authors:** Shuai Chen, Xiaofei Cao, Jinyang Zhang, Wanying Wu, Bing Zhang, Fangqing Zhao

**Affiliations:** ^1^ Beijing Institutes of Life Science Chinese Academy of Sciences Beijing 100101 China; ^2^ University of Chinese Academy of Sciences Beijing 100049 China; ^3^ Key Laboratory of Systems Biology Hangzhou Institute for Advanced Study University of Chinese Academy of Sciences Hangzhou 310013 China; ^4^ Center for Excellence in Animal Evolution and Genetics Chinese Academy of Sciences Kunming 650223 China

**Keywords:** CAPRIN1, circVAMP3, hepatocellular carcinoma, liquid–liquid phase separation

## Abstract

Previous studies have identified the regulatory roles of circular RNAs (circRNAs) in human cancers. However, the molecular mechanisms of circRNAs in hepatocellular carcinoma (HCC) remain largely unknown. This study screens the expression profile of circRNAs in HCC and identifies circVAMP3 as a significantly downregulated circRNA in HCC tissues. HCC patients with low circVAMP3 expression present poor prognosis. circVAMP3 negatively regulates the proliferation and metastasis of HCC cells in vitro and in vivo by driving phase separation of CAPRIN1 and promoting stress granule formation in cells, which can downregulate the protein level of Myc proto‐oncogene protein by inhibiting c‐Myc translation. Furthermore, circVAMP3 is widely expressed in many human tissues and is downregulated in related cancers. Therefore, circVAMP3 is a potential prognostic indicator for HCC and may serve as a therapeutic target for HCC treatment.

## Graphical abstract

A circular RNA, circVAMP3, is characterized here which is downregulated in hepatocellular carcinoma and other tumors. circVAMP3 interacts with CAPRIN1 and G3BP1 to trigger phase separation of CAPRIN1 and promote stress granule formation in cells by acting as a molecular scaffolder. Through stress granules, circVAMP3 exerts its tumor suppressor properties by inhibiting translation of c‐Myc.

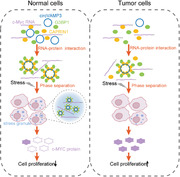

## 1 Introduction

Circular RNAs (circRNAs) are a class of closed‐loop RNAs generated from back‐splicing of pre‐mRNAs and often display cell type‐specific, tissue‐specific, or developmental stage‐specific expression pattern.^[^ ^1^, ^2^, ^3^, ^4^ ^]^ circRNAs have neither a 5’‐cap nor a 3’‐polyA tail; therefore, they exhibit RNase R‐resistant properties and are more stable than linear RNAs.^[^ ^5^ ^]^ In recent years, circRNAs have been reported to regulate various biological processes, including proliferation, aging, tissue development, and immune response.^[^ ^6^, ^7^, ^8^, ^9^ ^]^ Similar to other noncoding RNAs, circRNAs perform their molecular functions mainly by interacting with microRNAs or proteins.^[^ ^10^ ^]^ In addition, circRNAs may proceed with cap‐independent translation.^[^ ^11^ ^]^ Several studies have shown that circRNAs play regulatory roles in many human diseases,^[^ ^6^ ^]^ including cancer.

Hepatocellular carcinoma (HCC) is a prevalent malignant tumor with high morbidity and mortality rates. With 905 677 new cases and 830 180 deaths in 2020, primary liver cancer has become the sixth most diagnosed cancer and third deadly cancer worldwide.^[^ ^12^ ^]^ Due to the high recurrence and metastasis rates of HCC, the prognosis of patients remains poor even though treatment techniques for HCC are improving.^[^ ^13^, ^14^ ^]^ Hence, there is an urgent need to investigate the molecular pathological mechanisms and identify new diagnostic and therapeutic targets for HCC. Recently, numerous circRNAs have been reported to be deregulated in cancers and are associated with tumorigenesis and cancer progression.^[^ ^15^ ^]^ Nevertheless, understanding the molecular mechanisms of circRNAs is still limited. The most investigated molecular function of circRNAs in HCC is involved in microRNA sponge.^[^ ^16^ ^]^ For example, circMTO1 suppresses HCC progression by sponging miR‐9 and increasing p21 RNA expression.^[^ ^17^ ^]^ Some studies have reported that circRNAs can regulate the cell cycle and other processes by binding to proteins.^[^ ^18^, ^19^ ^]^ However, the protein binding role of circRNAs in HCC progression requires further study.

This study characterized a circRNA termed circVAMP3 from the RNA‐seq data of paired HCC and adjacent liver tissues. circVAMP3 was derived from back‐splicing of exon3 and exon4 of the VAMP3 gene. We found that circVAMP3 can suppress the proliferation and metastasis of HCC cells for the first time. circVAMP3 interacts with CAPRIN1 and drives the phase separation of CAPRIN1, thus promoting stress granule (SG) formation in cells. Moreover, circVAMP3, CAPRIN1, and c‐Myc colocalized into stress granules and the translation of c‐Myc was also inhibited under stress conditions. In addition, circVAMP3 is widely expressed in many human tissues and is downregulated in the corresponding tumor tissues. These findings unveil the role of the circVAMP3 in HCC progression and suggest that circVAMP3 may serve as a diagnostic target for HCC and other malignant tumors.

## 2 Results

### 2.1 circVAMP3 Is Decreased in HCC and Negatively Associated with Patient Prognosis

**Figure 1:**
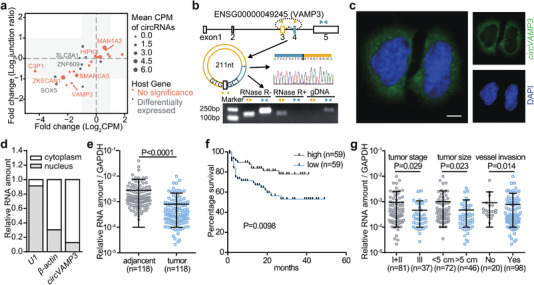
circVAMP3 is downregulated and associated with clinic outcomes in HCC patients. a) Expression level and junction ratio of significantly changed circRNAs in HCC and adjacent liver tissues. Each node represents a significantly expressed circRNA, with its size representing the mean expression level of the circRNA (measured by CPM) in HCC patients. The nodes are named by their host genes, with the node color showing whether the host genes are differentially expressed or not. b) Illustration of the genomic region of VAMP3 and the back‐splicing product of exon 3 and 4 (circVAMP3). Validation of head‐to‐tail splice junction site by PCR and sanger sequencing using the divergent primers (orange). Bottom, Polymerase chain reaction (PCR) of total RNA with or without RNase R treatment and genome DNA from SMMC‐7721 cells by convergent (blue) and divergent (orange) primers. c) RNA fluorescence in situ hybridization of circVAMP3 in SMMC‐7721 cells. Green region shows the distribution of circVAMP3 using antisense probe, blue region shows the nuclei staining by DAPI. Scale bar, 5 µm. d) Relative distribution of circVAMP3 in SMMC‐7721 cells determined by RT‐qPCR in different cell fractions. U1 was served as the nuclear RNA marker and *β*‐actin was served as the cytoplasmic RNA marker. e) RNA levels of circVAMP3 detected in randomly selected 118 pairs of HCC and adjacent normal tissues by qPCR (mean ± standard deviation, SD). The statistical significance was performed by two‐tailed paired Student *t*‐test. f) Kaplan–Meier survival analysis of the correlation between circVAMP3 level and overall survival in 118 HCC patients. Patients were interrupted for analysis by the median value. Statistical significance was performed by log‐rank test. g) Relative circVAMP3 expression in 118 HCC tumor samples, with tumor stage (I–II or III), with tumor size (< 5 or > 5), with or without vessel invasion. Data are expressed as mean ± SD and statistical significance was performed by chi‐square test.

To identify tumor‐associated circRNAs, we analyzed and screened differentially expressed circRNAs from sequencing data of ribosome RNA‐depleted total RNA from 20 pairs of HCC and adjacent tissues. We screened 17 circRNAs that were differentially expressed in abundance and junction ratio (back‐spliced junction reads divided by forward‐spliced junction reads), whereas the expression of their host mRNAs was not significantly changed (**Figure** **1**a). Among these candidates, circVAMP3 was selected for further functional studies, as it was unstudied and ranked the top among the significantly downregulated circRNAs in HCC tissues.

circVAMP3 was generated from the circularization of exon 3 and exon4 of the VAMP3 gene, which was located on chromosome 1 of the human genome (Figure 1b). To validate the transcripts of circVAMP3, we designed convergent and divergent primers to amplify the canonical or back‐spliced isoforms of VAMP3 RNA. The head‐to‐tail splice junction site was confirmed by polymerase chain reaction (PCR) and Sanger sequencing using divergent primers (Figure 1b). RNase R resistance analysis showed that circVAMP3, except VAMP3 mRNA, could be resistant to RNase R digestion (Figure 1b and Figure S1a, Supporting Information). Reverse transcription and quantitative PCR (RT‐qPCR) of cytoplasmic and nuclear circVAMP3 and fluorescence in situ hybridization (FISH) of circVAMP3 revealed that this circular transcript was mainly located in the cytoplasm (Figure 1c,d). Thereafter, to explore the relationship between circVAMP3 and HCC, we quantified the expression of circVAMP3 in 118 clinical samples of HCC patients using RT‐qPCR. The results showed that circVAMP3 was significantly downregulated in tumor tissues compared to adjacent normal tissues (Figure 1e). Kaplan–Meier survival analysis demonstrated that low circVAMP3 expression levels were associated with poor overall survival (*p* = 0.0098) (Figure 1f). Moreover, the expression of circVAMP3 was negatively correlated with tumor size and lower circVAMP3 levels exhibited a late tumor stage and tumor vessel invasion, respectively (Figure 1g and Table S2, Supporting Information). Therefore, these results strongly indicate that circVAMP3, formed by the circularization of the VAMP3 gene, is associated with poor survival of HCC patients.

### 2.2 circVAMP3 Inhibits HCC Cell Proliferation and Metastasis in Vitro and in Vivo

**Figure 2:**
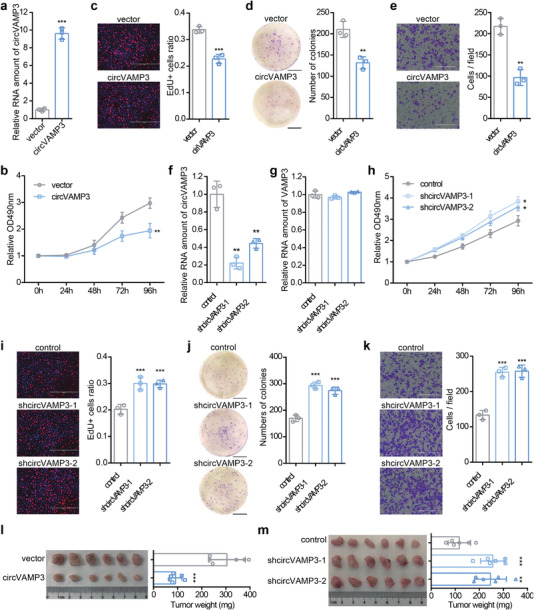
circVAMP3 inhibits SMMC‐7721 cell proliferation, growth and metastasis. a) Relative RNA level of circVAMP3 in SMMC‐7721 cells stably overexpressing circVAMP3 normalized to GAPDH. Cell proliferation of SMMC‐7721 cells stably overexpressing circVAMP3 using b) MTS assay and c) EdU staining assay. d) Colony formation assay of SMMC‐7721 cells stably overexpressing circVAMP3. e) Cell metastasis of SMMC‐7721 cells stably overexpressing circVAMP3 using transwell cell migration assay. Relative RNA level of f) circVAMP3 and g) VAMP3 in SMMC‐7721 cells stably silencing circVAMP3 normalized to GAPDH. Cell proliferation of SMMC‐7721 cells stably silencing circVAMP3 using h) MTS assay and i) EdU staining assay. j) Colony formation assay of SMMC‐7721 cells stably silencing circVAMP3. k) Cell metastasis of SMMC‐7721 cells stably silencing circVAMP3 using transwell cell migration assay. Data in (a)–(k) are presented as mean ± SD, *n* = 3. * *P* < 0.05, ** *P* < 0.01, *** *P* < 0.001 by two‐tailed unpaired Student *t*‐test. l,m) Dissected tumors and their weights from the nude mice that were subcutaneously injected with l) control or circVAMP3 overexpressed SMMC‐7721 cells and m) circVAMP3 silenced SMM‐C7721 cells. Data in (l) and (m) are presented as mean ± SD, *n* = 3. * *P* < 0.05, ** *P* < 0.01, *** *P* < 0.001 by two‐tailed unpaired Student t test. Scale bars in (c), (e), (i), and (k) are 400 µm; scale bars in (d) and (j) are 1 cm.

To further verify the carcinostatic role of circVAMP3 in HCC, we stably overexpressed and silenced circVAMP3 in HCC cell lines, SMMC‐7721, and Huh7. Overexpression of circVAMP3 was confirmed by RT‐qPCR and northern blot analysis in circVAMP3 expressing vector‐transfected cells compared with empty vector‐transfected cells (**Figure** **2**a and Figure S1b,c, Supporting Information). MTS assays, 5‐ethynyl‐2′‐deoxyuridine (EdU) staining assays, and colony formation assays demonstrated that cells overexpressing circVAMP3 exhibited decreased proliferation and growth capacities relative to cells transfected with empty vector (Figure 2b–d and Figure S1d–f, Supporting Information). In addition, transwell cell migration assays verified that cell migration ability was also significantly decreased in circVAMP3 overexpressing groups compared with the control groups (Figure 2e and Figure S1g, Supporting Information). In contrast, we stably knocked down circVAMP3 by two short hairpin RNAs (shRNAs) independently using a lentiviral vector in SMMC‐7721 and Huh7 cells. As verified by RT‐qPCR, circVAMP3 was significantly silenced in cells infected with lentivirus containing two junction site‐specific shRNA sequences, whereas VAMP3 mRNA levels did not change (Figure 2f,g, and Figure S1h,i, Supporting Information). Depletion of circVAMP3 significantly promoted cell proliferation, colony formation, and migration ability as demonstrated by MTS (3‐(4,5‐dimethylthiazol‐2‐yl)‐5‐(3‐carboxymethoxyphenyl)‐2‐(4‐sulfophenyl)‐2H‐tetrazolium) assays, EdU staining assays, colony formation assays, and transwell cell migration assays (Figure 2h–k and Figure S1j–m, Supporting Information).

To investigate the effects of circVAMP3 on HCC tumorigenesis in vivo, SMMC‐7721 cells stably overexpressing or depleting circVAMP3 were subcutaneously injected into nude mice. The weight and volume of the tumors developed from the overexpression group were dramatically reduced compared to those in the control group (Figure 2l and Figure S2a, Supporting Information). Cells with silenced circVAMP3 developed larger tumors than those in the control group (Figure 2m and Figure S2b, Supporting Information). Taken together, these results demonstrate that circVAMP3 acts as a carcinostatic substance in HCC cells both in vitro and in vivo.

### 2.3 circVAMP3 Interacts with CAPRIN1 and G3BP1 Protein in HCC Cells

**Figure 3:**
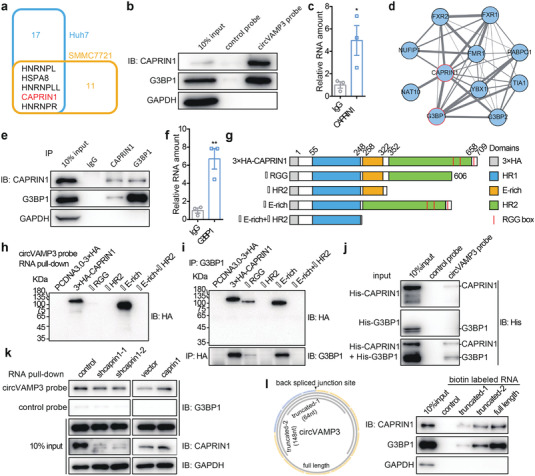
circVAMP3 interacts with CAPRIN1 and G3BP1 protein in Huh7 and SMMC‐7721 cells. a) Venn diagram of circVAMP3 binding proteins in Huh7 and SMMC‐7721 cells detected by RNA pull‐down and mass spectrometry. b) Immunoblotting analysis of CAPRIN1 and G3BP1 in RNA pull‐down samples by circVAMP3 probes and control probes in SMMC‐7721 cells. c) RIP analysis of circVAMP3 enriched by CAPRIN1 in SMMC‐7721 cells. Data are expressed as mean ± SD, *n* = 3. * *P* < 0.05 by two‐tailed unpaired Student *t*‐test. d) Protein–protein interaction network of CAPRIN1 and other proteins. Thickness of edges represents the interacting scores. e) Co‐IP followed by immunoblotting analysis of CAPRIN1 and G3BP1 in SMMC‐7721 cells. f) RIP analysis of circVAMP3 enriched by G3BP1 in SMMC‐7721 cells. Data are expressed as mean ± SD, *n* = 3. ** *P* <0.01 by two‐tailed unpaired Student *t*‐test. g) Deletion mapping of the domains in CAPRIN1. h) Immunoblotting analysis of truncated 3 × HA‐tagged CAPRIN1 proteins in RNA pull‐down samples by circVAMP3 probes. i) Co‐IP and immunoblotting analysis of truncated 3 × HA‐tagged CAPRIN1 and G3BP1 in SMMC‐7721 cells. j) Immunoblotting analysis of purified 6 × His‐tagged CAPRIN1 and 6 × His‐tagged G3BP1 proteins in RNA pull‐down samples by circVAMP3 probs. k) Immunoblotting analysis of CAPRIN1 and G3BP1 in RNA pull‐down samples by circVAMP3 probes in CAPRIN1 depleted and overexpressed SMMC‐7721 cells. l) Deletion mapping of circVAMP3 RNAs and immunoblotting analysis of CAPRIN1 and G3BP1 in RNA pull‐down samples by biotin‐labeled full‐length or truncated circVAMP3.

To elucidate the molecular mechanism of the carcinostatic role of circVAMP3 in HCC, we performed a biotin‐labeled RNA pull‐down assay using circular junction targeted probes followed by mass spectrometry (MS) in SMMC‐7721 and Huh7 cells. RT‐qPCR of circVAMP3 and VAMP3 in RNA pull‐down precipitate showed that circVAMP3, except for VAMP3 mRNA, was highly enriched using a junction‐specific probe compared with the control probe (Figure S3a,b, Supporting Information). After detecting the RNA pull‐down precipitate by sodium dodecyl sulfate‐polyacrylamide gel electrophoresis (SDS‐PAGE) analysis, followed by Coomassie Brilliant Blue R250 staining, we found certain specific protein bands in the circVAMP3 pull‐down precipitate (Figure S3c, Supporting Information). From these protein bands, we identified 22 proteins in Huh7 cells and 16 proteins in SMMC‐7721 cells by MS analysis (**Figure** **3**a), with five proteins shared by the two cell lines (Figure 3a and Table S3, Supporting Information). Considering that circVAMP3 was located mainly in cytoplasm, we performed RNA pull down from SMMC‐7721 and Huh7 cytoplasmic lysates using circVAMP3 probe. Western blot analysis showed that among the five proteins, only CAPRIN1 could be highly enriched by circVAMP3 probe from both SMMC‐7721 and Huh7 cells (Figure S3d,e, Supporting Information). Western blot analysis of whole cell lysates also confirmed that CAPRIN1 could be pulled down by the circVAMP3 probe instead of the control probe (Figure 3b). Moreover, the RNA‐binding protein immunoprecipitation (RIP) assay demonstrated that the antibodies of CAPRIN1 could significantly enrich circVAMP3 relative to IgG antibodies (Figure 3c). To further explore the role of CAPRIN1 in the tumor suppression process by circVAMP3, we analyzed the interaction network of CAPRIN1 and other proteins using the Search Tool for the Retrieval of Interacting Genes/Proteins (STRING) database.^[^ ^20^ ^]^ The network showed that G3BP1 protein had the highest correlation with CAPRIN1 (Figure 3d and Figure S3f, Supporting Information). The interaction between CAPRIN1 and G3BP1 was supported by a coimmunoprecipitation (co‐IP) assay and western blot analysis (Figure 3e). Furthermore, the interaction between circVAMP3 and G3BP1 was verified by RNA pull‐down and RIP assays (Figure 3b,f). These findings demonstrate that circVAMP3 specifically interacts with CAPRIN1 and G3BP1 in HCC cells.

### 2.4 circVAMP3 Binds to the Complex of CAPRIN1 and G3BP1 by Directly Interacting with CAPRIN1

To obtain insights into the interaction patterns among circVAMP3, CAPRIN1, and G3BP1, the 3 × hemagglutinin (HA)‐tagged CAPRIN1 protein was expressed in SMMC‐7721 cells and a series of truncated forms of CAPRIN1 based on its domain structure were constructed (Figure 3g and Figure S3g, Supporting Information). RNA pull‐down assay showed that the RGG box (607–709 amino acids) of CAPRIN1 was responsible for circVAMP3 binding (Figure 3h). The co‐IP assay indicated that the N‐terminal of the KH2 domain (352–605 amino acids that do not contain the RGG box) of CAPRIN1 was essential for the interaction with G3BP1 (Figure 3i). As a result, circVAMP3 and G3BP1 bind to different parts of CAPRIN1. In addition, to investigate whether circVAMP3 can directly interact with CAPRIN1 and G3BP1, in vitro binding experiments were performed using in vitro transcribed circVAMP3 and purified 6 × His‐tagged CAPRIN1 and G3BP1. RNA pull‐down assay showed that compared with precipitates in the control group, CAPRIN1 was detected in the complex pulled‐down by the cricVAMP3 probe, whereas G3BP1 was only detected in the presence of CAPRIN1. This result indicates that CAPRIN1 can bind to circVAMP3 directly, while G3BP1 interacts with circVAMP3 only in the presence of CAPRIN1 (Figure 3j). CAPRIN1 knockdown inhibited the interaction between circVAMP3 and G3BP1, whereas CAPRIN1 overexpression enhanced this interaction (Figure 3k and Figure S3h,i, Supporting Information). In addition, longer circVAMP3 transcript fragments could bind more CAPRIN1 and G3BP1 proteins (Figure 3l and Figure S3j, Supporting Information), indicating a potential scaffolding role of circVAMP3. Collectively, these findings demonstrate that circVAMP3 interacts with the CAPRIN1‐G3BP1 complex by binding to CAPRIN1.

### 2.5 CAPRIN1 and G3BP1 Can Form Granules and Exhibit Liquid‐Like Features

**Figure 4:**
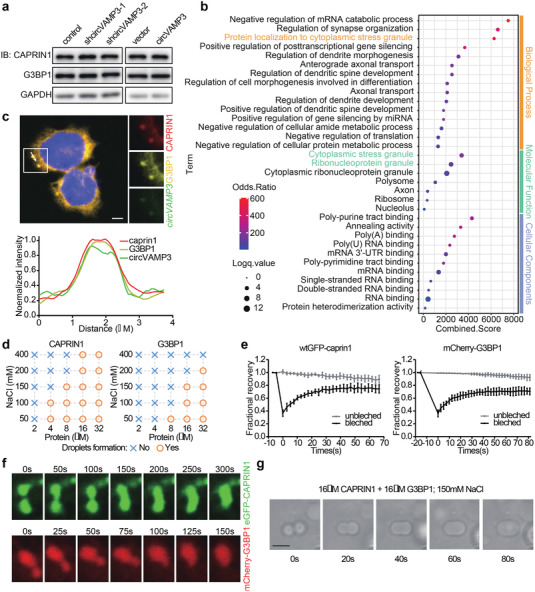
CAPRIN1 and G3BP1 can undergo phase separation in vitro and in vivo. a) Immunoblotting analysis of CAPRIN1 and G3BP1 in circVAMP3 silenced and overexpressed SMMC‐7721 cells. b) Gene Ontology enrichment analysis of the genes from the CAPRIN1 interaction network. c) Top: RNA fluorescence in situ hybridization and immunofluorescence analysis of circVAMP3, CAPRIN1 and G3BP1 in SMMC7721 cells, nuclei are stained with DAPI. Scale bar, 5 µm. Bottom: curves of fluorescence intensities of the position marked by white arrow in the merged image. d) Diagrams of phase separation behaviors of different concentrations of purified CAPRIN1 (left) and G3BP1 (right) in different concentrations of NaCl (with 1% Dextran T500). e) Fluorescence recovery of wtGFP‐CAPRIN1 and mCherry‐G3BP1 condensates expressed in SMMC‐7721 cells after photobleaching. Data are presented as mean ± SD; unbleached, *n* = 3; bleached, n = 3. f) Images of fusion progress of enhanced GFP (EGFP)‐CAPRIN1 (top) and mCherry‐G3BP1 (bottom) condensates expressed in SMMC‐7721 cells in different time points. g) Images of fusion progress of purified CAPRIN1 and G3BP1 protein droplets in different time points. Scale bar, 20 µm.

Considering that circVAMP3 interacts with the CAPRIN1‐G3BP1 complex, we further investigated the functional interplay of these molecules. Western blot analysis of CAPRIN1 and G3BP1 in circVAMP3 silenced or overexpressed cells revealed that circVAMP3 did not affect the protein levels of CAPRIN1 and G3BP1 (**Figure** **4**a and Figure S4a,b, Supporting Information). Gene Ontology (GO) enrichment analysis performed using Enrichr web server^[^ ^21^, ^22^ ^]^ showed that CAPRIN1 and its interacting proteins were involved in cytoplasmic granules (Figure 4b). RNA FISH of circVAMP3 combined with immunofluorescence (IF) of CAPRIN1 and G3BP1 proved that circVAMP3, CAPRIN1, and G3BP1 were localized in the cytoplasm and colocalized in condensates (Figure 4c). In addition, previous studies have implicated that G3BP1 plays a key role in SG formation,^[^ ^23^, ^24^ ^]^ whereas CAPRIN1 is colocalized in many common cytoplasmic condensates such as SGs, P‐bodies, and neuronal granules.^[^ ^25^, ^26^, ^27^ ^]^ To examine whether the complex of circVAMP3, CAPRIN1, and G3BP1 form cytoplasmic granules, in vitro and in vivo experiments were performed. We constructed green fluorescent protein (GFP)‐fused CAPRIN1 and mCherry‐fused G3BP1 expressing vectors and expressed them in SMMC‐7721 cells (Figure S5a, Supporting Information). Both GFP‐CAPRIN1and mCherry‐G3BP1 formed granules that were colocalized in the cytoplasm (Figure S5b, Supporting Information). Furthermore, under the condition of physiological salt concentration (150 × 10^−3^ m NaCl), the purified CAPRIN1 formed droplets at concentrations of 32 × 10^−6^ m or more (Figure S5c, Supporting Information). However, we could not observe the droplet formation of purified G3BP1 over a range of protein concentrations (Figure S5c, Supporting Information). This observation was supported by a previous research.^[^ ^23^ ^]^ The droplet formation capability of CAPRIN1 and G3BP1 was enhanced by adding the crowding agent Dextran T500 (Figure 4d and Figure S5d,e, Supporting Information). In addition, increased protein concentration and decreased NaCl concentration promoted droplet formation of the two proteins (Figure 4d and Figure S5d,e, Supporting Information). To further verify the liquid‐like properties of these granules, we performed fluorescence recovery after photobleaching (FRAP) assay in wtGFP‐CAPRIN1‐and mCherry‐G3BP1 expressed cells. Photobleaching of a region of each wtGFP‐CAPRIN1 or mCherry‐G3BP1 granules resulted in a rapid recovery of fluorescence, whereas there was no significant change in fluorescence in unbleached granules (Figure 4e and Figure S5f, Supporting Information). Moreover, we observed that small granules could fuse to form larger granules in SMMC‐7721 cells (Figure 4e), and small droplets of the two proteins could fuse to form larger droplets in vitro (Figure 4f). Overall, these results suggest that CAPRIN1 and G3BP1 can be assembled into liquid‐like granules in vitro and in vivo, and that circVAMP3 colocalizes in the granules.

### 2.6 circVAMP3 Promotes the Formation of CAPRIN1 and G3BP1 Based Granules

**Figure 5:**
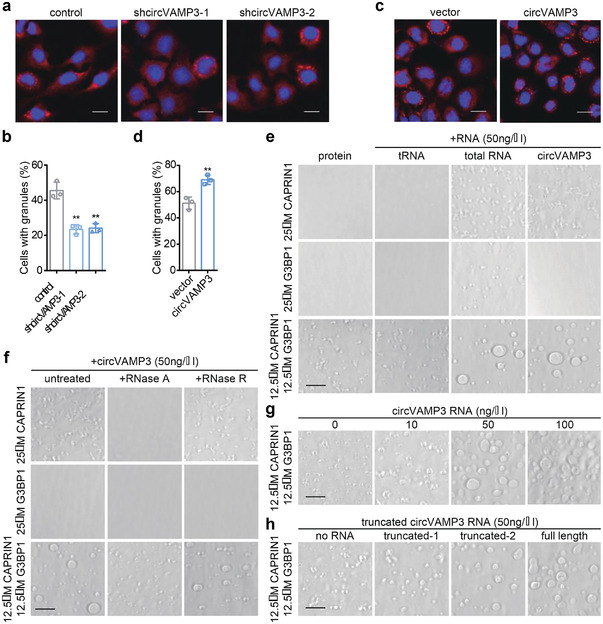
circVAMP3 triggers phase separation of CAPRIN1 and promotes stress granule formation. a) Immunofluorescence of caprin1 in control and circVAMP3 silenced SMMC7721 cells treated with sodium arsenite (100 × 10^−6^ m, 40 min). Scale bar, 20 µm. b) The percentage of control and circVAMP3 silenced cells with stress granules. c) Immunofluorescence of caprin1 in control and circVAMP3 overexpressed SMMC7721 cells treated with sodium arsenite (100 × 10^−6^ m, 40 min). Scale bar, 20 µm. d) The percentage of control and circVAMP3 overexpressed cells with stress granules. e) Phase separation of purified CAPRIN1 and G3BP1 with or without addition of 50 ng *μ*L^−1^ different kinds of RNAs in the buffer containing 150 × 10^−3^ m NaCl. f) Phase separation of purified CAPRIN1 and G3BP1 with 50 ng *μ*L^−1^ circVAMP3 with or without addition of 10 µg mL^−1^ RNaseA or 1 U *μ*L^−1^ RNaseR. g) Phase separation of purified CAPRIN1 and G3BP1 with addition of different concentration of circVAMP3 RNA. h) Phase separation of purified CAPRIN1 and G3BP1 with addition of different truncated circVAMP3 RNA fragments. Images of samples in (e)–(h) were acquired immediately after incubating at room temperature for 10 min. Data in (b) and (d) are expressed as mean ± SD, *n* = 3. *** *P* < 0.001. Scale bars in (e)–(h) are 20 µm.

Considering that circVAMP3 colocalizes with CAPRIN1 and G3BP1 condensates, we next investigated whether cricVAMP3 could induce the formation of G3BP1‐composed SGs. We first induced SGs by stimulation with sodium arsenite in circVAMP3 stably silenced or overexpressed SMMC‐7721 cells. We found that the ratio of SG‐containing cells was significantly increased in circVAMP3 overexpressed cells and decreased in circVAMP3 silenced cells (**Figure** **5**a–d). In addition, to identify the influence of circVAMP3 on droplet formation of CAPRIN1 and G3BP1 in vitro, we purified total RNA from SMMC‐7721 cells and transcribed and cyclized circVAMP3 RNA in vitro and incubated them with CAPRIN1 or G3BP1 proteins without Dextran T500, respectively. In contrast to tRNA, total RNA could promote droplets of CAPRIN1, G3BP1, and the mixture of CAPRIN1 and G3BP1. However, circVAMP3 induced droplets of CAPRIN1 and the mixture of CAPRIN1 and G3BP1, but failed to induce droplets of sole G3BP1 (Figure 5e and Figure S6a, Supporting Information). Moreover, circVAMP3‐triggered droplets of CAPRIN1 and G3BP1 could be reversed by RNase A instead of RNase R (Figure 5f and Figure S6b, Supporting Information), indicating that the droplets were indeed triggered by this circular transcript. To determine whether the droplet formation of CAPRIN1 and G3BP1 was affected by the concentration of circVAMP3, we incubated CAPRIN1 and G3BP1 with increasing concentrations of circVAMP3. A positive correlation was observed between circVAMP3 concentration and the droplet formation ability of CAPRIN1 and G3BP1 (Figure 5g and Figure S6c, Supporting Information). In addition, longer fragments of circVAMP3 transcripts had a stronger ability to drive droplet formation of CAPRIN1 and G3BP1 (Figure 5h and Figure S6d, Supporting Information). These findings suggest that circVAMP3 promotes the formation of CAPRIN1 and G3BP1 SGs in a length‐and concentration‐dependent manner and that CAPRIN1 is essential in this process.

### 2.7 circVAMP3 Exerts Tumor Suppressor Properties by Inhibiting Translation of c‐Myc

**Figure 6:**
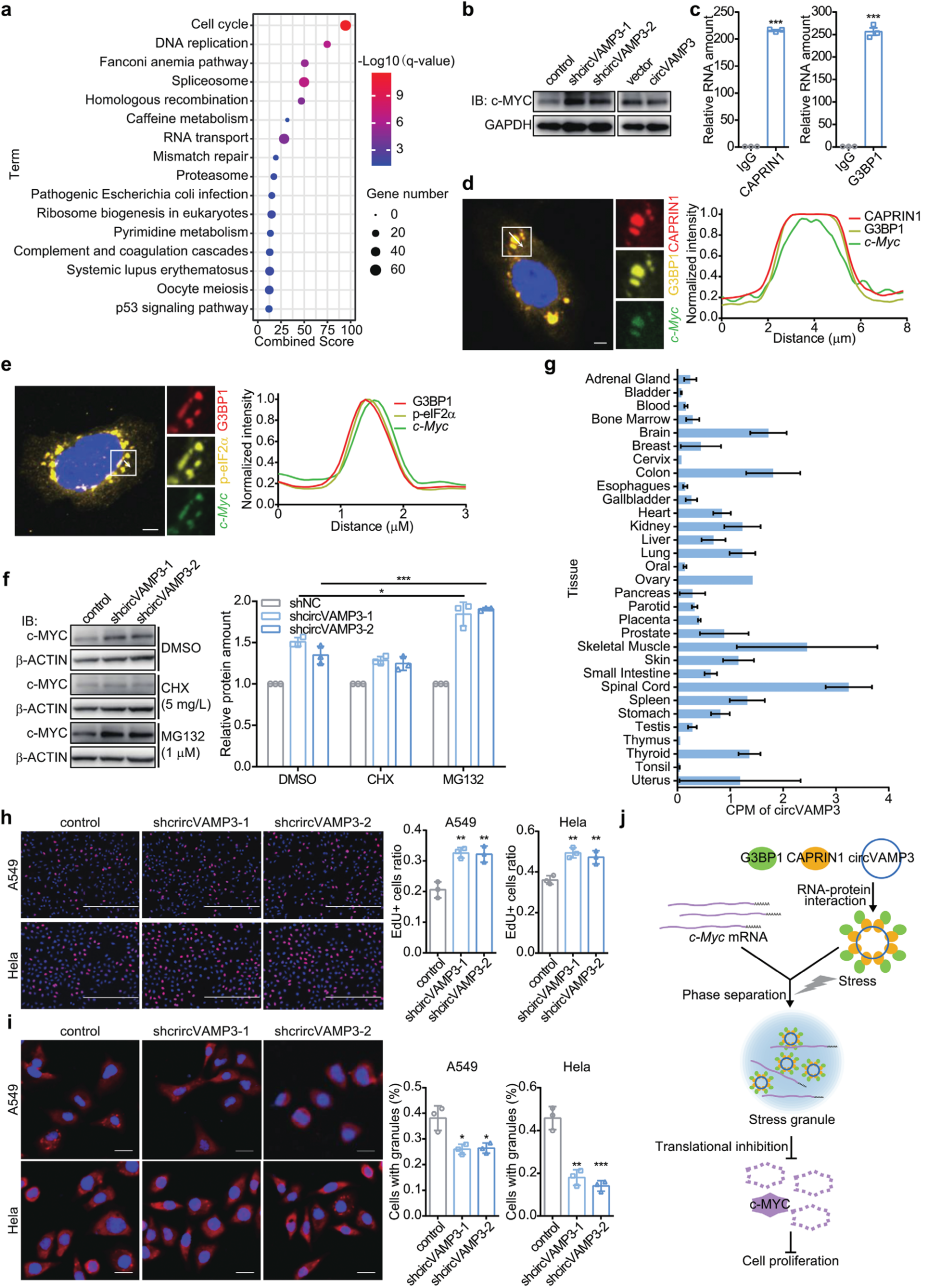
circVAMP3 inhibits the translation of c‐Myc and exerts its tumor suppressor function in other cancers. a) KEGG pathway analysis of differentially expressed genes in HCC and adjacent tissues. b) Immunoblotting analysis of c‐MYC in circVAMP3 silenced and overexpressed SMMC‐7721 cells. c) RIP analysis of c‐Myc enriched by CAPRIN1 and G3BP1 in SMMC‐7721 cells. d) Left: RNA fluorescence in situ hybridization and immunofluorescence analysis of c‐Myc, CAPRIN1 and G3BP1 in SMMC7721 cells treated with sodium arsenite (100 × 10^−6^ m, 40 min), nuclei are stained with DAPI. Scale bar, 5 µm. Right: Curves of fluorescence intensities of the position marked by white arrow in the merged image. e) Left: RNA fluorescence in situ hybridization and immunofluorescence analysis of c‐Myc, p‐eIF2*α* and G3BP1 in SMMC7721 cells treated with sodium arsenite (100 × 10^−6^ m, 40 min), nuclei are stained with DAPI. Scale bar, 5 µm. Right: curves of fluorescence intensities of the position marked by white arrow in the merged image. f) Left: Immunoblotting analysis of c‐MYC in control and circVAMP3 silenced SMMC‐7721 cells treated with sodium arsenite (100 × 10^−6^ m, 40 min) together with CHX (5 mg L^−1^, 1 h) or MG132 (1 × 10^−6^ m, 1 h). Right: Relative protein levels of c‐MYC. g) Expression level of circVAMP3 in various human tissues. Data are expressed as mean ± standard error of the mean (SEM). h) Cell proliferation of control and circVAMP3 silenced Hela and A549 cells using EdU staining assay. Scale bar, 400 µm. i) Left: Immunofluorescence of caprin1 in control and circVAMP3 silenced Hela and A549 cells treated with sodium arsenite (100 × 10^−6^ m for Hela and 500 × 10^−6^ m for A549, 40 min). Scale bar, 20 µm. Right: The percentage of cells with stress granules. j) The model of the effects of circVAMP3 on SGs formation cell proliferation. Data in (c), (f), (h), and (i) are expressed as mean ± SD, *n* = 3. * *P* < 0.05, ** *P* < 0.01, *** *P* < 0.001 by two‐tailed unpaired Student *t*‐test.

Given the effect of circVAMP3 on the induction of SGs, we assumed that circVAMP3 may exert its tumor suppressor function in HCC through the granules. Kyoto Encyclopedia of Genes and Genomes (KEGG) pathway analysis of differentially expressed genes in 20 pairs of HCC and adjacent tissues using Enrichr web server^[^ ^21^, ^22^ ^]^ showed that cell cycle progression was significantly enriched (**Figure** **6**a). To explore the role of circVAMP3 in cell cycle progression, we first investigated the expression of several well‐studied proteins that play important roles in the progression of HCC cells. Western blot analysis showed that the expression of c‐MYC protein was upregulated in circVAMP3 silenced SMMC‐7721 cells and downregulated in circVAMP3 overexpressed SMMC‐7721 cells, while c‐Myc mRNA expression level was not significantly changed in circVAMP3 silenced SMMC‐7721 cells and upregulated in circVAMP3 overexpressed SMMC‐7721 cells (Figure 6b and Figure S4c and Figure S7a, Supporting Information). These results suggest that the differential expression of c‐MYC protein was not due to the influence of circVAMP3 on c‐Myc gene expression. Immunofluorescence analysis of c‐MYC and CAPRIN1 revealed that c‐MYC protein was not colocalized in SGs (Figure S7b, Supporting Information). However, RIP assay followed by RT‐qPCR analysis revealed that CAPRIN1 and G3BP1 proteins pulled down c‐Myc mRNA (Figure 6c). Moreover, RNA FISH of c‐Myc combined with immunofluorescence of CAPRIN1 and G3BP1 in sodium arsenite‐treated SMMC‐7721 cells showed that c‐Myc RNA, CAPRIN1, and G3BP1 were colocalized in SGs (Figure 6d). Previous studies found that phase separation of CAPRIN1 may regulate mRNA translation,^[^ ^28^, ^29^ ^]^ and under stress conditions, the formation of SGs is involved in the translational inhibition of many mRNAs.^[^ ^30^ ^]^ Therefore, we speculated that circVAMP3 may affect the expression level of c‐MYC protein by regulating c‐Myc translation. RNA FISH and immunofluorescence revealed that the translation of c‐Myc was inhibited in sodium arsenite‐treated SMMC‐7721 cells, as c‐Myc RNA was colocalized with phosphorylated eIF2*α*, which functions as an inhibitor for translation initiation (Figure 6e). Moreover, after inhibiting the degradation of proteins by treatment with MG132, the ratio of c‐Myc in circVAMP3 depleted cells to control cells was significantly higher than that in cells treated with dimethyl sulfoxide (DMSO); however, the ratio was not significantly changed in cycloheximide (CHX)‐treated cells (Figure 6f). These findings indicate that the translation of c‐Myc can be inhibited by circVAMP3 under stress conditions.

### 2.8 A Potentially Universal Repression Role of circVAMP3 in Various Tumors

As most circRNAs exhibit tissue‐specific expression,^[^ ^3^, ^4^ ^]^ we explored the expression of circVAMP3 in various tissues derived from the circAtlas and MiOncoCirc database.^[^ ^31^, ^32^ ^]^ In contrast to the vast majority of circRNAs, circVAMP3 was widely expressed in various tissues, with the spinal cord, skeletal muscle, and colon ranking the top three highly expressed tissues (Figure 6g). Furthermore, circVAMP3 was downregulated in many types of tumors compared with their corresponding normal tissues (Figure S8a, Supporting Information). To verify the carcinostatic impact of circVAMP3 on other tumors, we stably silenced and overexpressed circVAMP3 in HeLa and A549 cells derived from human cervicarcinoma and pulmonary carcinoma, respectively (Figure S8b,c, Supporting Information). Knockdown of circVAMP3 in HeLa and A549 cells significantly promoted cell proliferation (Figure 6h). Moreover, under the stress conditions induced by sodium arsenite, silencing of circVAMP3 inhibited SG formation in HeLa and A549 cells (Figure 6i). On the contrary, overexpression of circVAMP3 significantly inhibited cell proliferation and promoted SG formation in Hela and A549 cells (Figure S8d,e, Supporting Information). Therefore, these findings strongly suggest that besides HCC, circVAMP3 may play a carcinostatic role and contribute to SG formation in various tumors.

## 3 Discussion

In this study, we identified a novel HCC‐related circRNA, circVAMP3, which is derived from exon3 and exon4 of the VAMP3 gene. Reduced circVAMP3 expression was correlated with poor prognosis of HCC patients, indicating that circVAMP3 may serve as a prognostic biomarker for HCC. Functional assays indicated that circVAMP3 suppresses the tumorigenicity of HCC cells. We further revealed that circVAMP3 inhibits HCC by interacting with the complex of CAPRIN1 and G3BP1 to drive SG formation and inhibit translation of the proto‐oncogene c‐Myc under stress conditions (Figure 6j). We found that circVAMP3 is widely expressed in various human tissues and is usually downregulated in the corresponding tumor tissues, indicating that it may play a universal role in regulating tumorigenesis. Cellular functional studies further support this speculation, as circVAMP3 inhibits proliferation and enhances SG formation in cervical cancer and lung cancer cells.

CircRNAs have been reported to play crucial roles in human diseases, including cardiovascular diseases, neurodegenerative diseases, and cancers.^[^ ^6^, ^33^, ^34^, ^35^ ^]^ In cancer biology, circRNAs serve as tumor suppressors or oncogenic drivers through a series of biological functions. The most investigated are microRNA sponge functions.^[^ ^36^, ^37^, ^38^ ^]^ Moreover, circRNAs may interact with RNA‐binding proteins to enhance or suppress their functions by sponging proteins,^[^ ^39^ ^]^ enhancing protein interactions^[^ ^18^ ^]^ or affecting the localization of proteins.^[^ ^40^ ^]^ In addition, circRNAs can be translated into proteins via a cap‐independent manner.^[^ ^41^, ^42^, ^43^, ^44^ ^]^ This study demonstrated for the first time that circRNAs can act as tumor suppressors by interacting with key components of SGs through liquid–liquid phase separation (LLPS).

LLPS occurs when proteins or RNAs accumulate in high‐concentration membrane‐less structures, which are separated from a soluble phase and exhibit liquid‐like properties. Protein interacting domains or intrinsically disordered regions that promote the interaction between proteins can trigger LLPS.^[^ ^45^, ^46^, ^47^ ^]^ RNAs may also play an essential role in the regulation of LLPS. For example, recent studies have found that lncRNA NEAT 1 can facilitate paraspeckles formation through scaffolding paraspeckle proteins such as NONO and SFPQ.^[^ ^48^, ^49^, ^50^ ^]^ Similarly, some RNAs act as scaffolders to promote LLPS of YTHDF proteins through m^6^A‐modification.^[^ ^51^, ^52^ ^]^ This study provides the first evidence that circRNA can trigger LLPS. We found that multiple CAPRIN1 proteins are bound to various sites of circVAMP3 through their conserved RGG domain, indicating that circVAMP3 can promote LLPS by scaffolding CAPRIN1 proteins. In addition, G3BP proteins can also undergo LLPS and serve as the core elements of SGs in cells.^[^ ^23^, ^24^ ^]^ However, as circVAMP3 does not directly interact with G3BP1, the in vitro LLPS of G3BP1 cannot be facilitated by circVAMP3. Considering that G3BP1 can interact with CAPRIN1 to form protein complexes, circVAMP3 may scaffold and concentrate the CAPRIN1‐G3BP1 complex by interacting with CAPRIN1, thus triggering LLPS. As circVAMP3 and VAMP3 liner transcripts share the same sequence (exon3 and 4), VAMP3 RNA may also have potential to drive LLPS. However, circVAMP3 are more stable than VAMP3 liner transcripts (Figure 1b), which exhibits its advantages in promoting LLPS.

SGs are cytoplasmic membrane‐less ribonucleoprotein (RNP) condensates that are formed in response to various environmental stresses such as heat shock, oxidative stress, osmotic stress, or nutrient starvation.^[^ ^53^, ^54^, ^55^ ^]^ SGs are also associated with RNA translation.^[^ ^54^, ^56^ ^]^ It has been reported that GIRGL lncRNA interacts with GLS1 mRNA and CAPRIN1 to suppress the translation of GLS1 mRNA by driving the formation of SGs.^[^ ^29^ ^]^ Moreover, phase separation of CAPRIN1 and FMRP modulates the in vitro translation and deadenylation of target RNAs.^[^ ^28^ ^]^ These studies highlight the translational regulatory role of LLPS in CAPRIN1. In this study, we observed that circVAMP3 inhibited the expression of the MYC proto‐oncogene protein by suppressing the translation of c‐Myc. c‐Myc mRNA also interacts with CAPRIN1 and G3BP1 and colocalizes with SGs under stress conditions.^[^ ^57^ ^]^ These findings indicate that circVAMP3 may regulate c‐Myc translation via SGs.

Similar to other noncoding RNAs, most circRNAs exhibit strong tissue‐specific expression, implying their specific function in various tissues.^[^ ^58^ ^]^ However, a very small number of circRNAs have been found to be widely expressed in many human tissues and have been proven to be functionally significant. For instance, a famous circRNA, circHIPK3, was abundant in many human tissues, including the brain, lung, heart, liver, stomach, and colon, and could serve as a diagnostic target.^[^ ^36^ ^]^ Another circRNA, circITCH was also expressed in various human tissues and acted as a tumor suppressor in lung cancer, HCC, breast cancer, ovarian cancer, osteosarcoma, glioma, and many other malignant tumors.^[^ ^59^ ^]^ In this study, we found that circVAMP3 was also a universally expressed circRNA in a vast majority of human tissues, which exhibited a significantly reduced expression in various tumor tissues compared with their corresponding normal human tissues, suggesting the universality of the tumor suppressor role of circVAMP3. Considering that circRNAs are more stable and exhibit longer half‐lives than linear RNAs due to their closed loop structure, circVAMP3 can serve as an ideal diagnostic and prognostic biomarker for many malignant tumors.

In conclusion, our study identified and characterized an important circRNA, circVAMP3, which can physically interact with CAPRIN1 to promote SG formation and inhibit c‐Myc translation. Considering that circVAMP3 is abundant in various human tissues and downregulated in their corresponding tumor tissues, it may serve as a novel diagnostic and therapeutic target for HCC and many other cancers.

## 4 Experimental Section

### mRNA and circRNA Analysis of HCC RNA‐seq Data

RNA‐seq data of HCC tumors and paired normal tissues from 20 HCC patients were obtained from the National Center for Biotechnology Information (NCBI) under accession number SRP069212.^[^ ^60^ ^]^ The human reference genome and gene annotation (Release 19, GRCh37.p13) were downloaded from the GENCODE website. For gene expression analysis, sequencing reads were aligned to the reference genome using HISAT2^[^ ^61^ ^]^ (v2.1.0) with the “–dta” option, and gene expression levels were estimated using StringTie^[^ ^62^ ^]^ (v1.3.3) with the “‐e” parameter. Differential expression analysis was performed using edgeR^[^ ^63^ ^]^ (v3.26.8) with the glm approach, and an adjusted *p*‐value threshold of 0.05, was applied to filter differentially expressed genes.

For circRNA analysis, back‐spliced junction sites were first identified using BWA‐mem^[^ ^64^ ^]^ (v0.7.17) and CIRI2^[^ ^65^ ^]^ (v2.0.6) pipeline. Then, CIRIquant^[^ ^66^ ^]^ (v1.0) was used for quantification and differential expression analysis. The expression level of circRNAs was measured using counts per million (CPM), and the logarithm fold‐change of CPM and junction ratio was calculated to measure the change in circRNA expression between tumor and adjacent samples.

The expression patterns of circVAMP3 in normal human tissues and tumor samples were downloaded from the circAtlas^[^ ^31^ ^]^ (https://circatlas.biols.ac.cn/) and MiOncoCirc^[^ ^32^ ^]^ (https://mioncocirc.github.io/) databases. The expression levels of circRNAs were measured using CPM to normalize the data size in different samples.

### Clinical Samples

Tumor and matched adjacent samples from 118 HCC patients were collected from Beijing 302 Hospital between February 2014 and March 2016. Clinical information of patients is shown in Table S2 in the Supporting Information. The HCC tissues and clinicopathological features were confirmed by pathologists. Informed consent was obtained from all patients. All studies were approved by The Ethics Committee of Beijing 302 Hospital.

### Cell Culture and Transfection

HEK293T, SMMC‐7721, Huh7, HeLa, and A549 cells were cultured in Dulbecco's modified Eagle's medium (Gibco, USA) containing 10% fetal bovine serum (Gibco, USA) and 1% penicillin‐streptomycin (Gibco, USA) at 37 °C in an incubator containing 5% CO_2_. Transfection of cells with plasmids was performed using HieffTrans Liposomal Transfection Reagent (YEASEN, China) according to the manufacturer's protocol.

### RNA Isolation, Reverse Transcription, and PCR

Total RNA was isolated using TRIzol reagent (Thermo Fisher Scientific, USA). Reverse transcription was performed with random hexamers using a FastKing RT kit (TIANGEN, China) according to the manufacturer's protocol. PCR was performed using PrimeSTAR Max DNA Polymerase (Takara, Japan). PCR products were detected using 1% agarose gel and Sanger sequencing. Quantitative real‐time PCR (RT‐qPCR) was performed using Hieff qPCR SYBR Green Master Mix (YEASEN, China) in a StepOne Plus Real‐time PCR system (Applied Biosystems, USA). The primers used for RT‐qPCR are listed in Table S4 in the Supporting Information. The relative transcript levels were analyzed by the comparative Ct method.

### RNase R Resistance Analysis

Total RNA (2 µg) from SMMC‐7721 cells was treated for 10 min at 37 °C with ribonuclease R (epicentral, USA) according to the manufacturer's instructions. For controls, 2 µg of total RNA was mock treated under the same conditions without the enzyme. After the treatment, the RNAs were purified using the TRIzol reagent (Thermo Fisher Scientific, USA). Reverse transcription of the products was then performed. The circular RNAs or linear RNAs were detected by PCR using divergent or convergent primers over 30 cycles followed by agarose gel electrophoresis.

### Cytoplasmic and Nuclear RNA Analysis

Cytoplasmic and nuclear fractions were extracted using the Nuclear and Cytoplasmic Protein Extraction Kit (Beyotime, China) according to the manufacturer's instructions. RNA was extracted from fractions. The localization of circVAMP3 was analyzed by RT‐qPCR. U1 served as the nuclear RNA marker, and *β*‐actin served as the cytoplasmic RNA marker. The primers used for RT‐qPCR are listed in Table S4 in the Supporting Information.

### FISH

RNA FISH was performed using a specific probe in the back‐splice region of circVAMP3 RNA. The sense and antisense fused sequences of the T7 promoter and probe sequences were synthesized and annealed. The DNA sequences are presented in Table S4 in the Supporting Information. Biotin‐labeled RNA probes were transcribed from the annealed products using a biotin RNA labeling mix (Roche, Switzerland) and HiScribe T7 Quick High Yield RNA Synthesis Kit (NEB, USA) according to the manufacturers’ protocols. Cells were grown to 60–80% confluence and then fixed with 4% paraformaldehyde. The prehybridization and hybridization experiments were performed using the Fluorescent In Situ Hybridization Kit (Ribobio, China) according to the manufacturer's protocol. After hybridization, cells were blocked with 5% bovine serum albumin (BSA) in 1 × phosphate buffered saline (PBS). Then cells were incubated with fluorescein isothiocyanate (FITC)‐labeled antibiotin antibody (Abcam, UK) for 2 h at room temperature, followed by three washes with 1 × PBS. The nucleus was then stained by 4′,6‐diamidino‐2‐phenylindole (DAPI). The cell slices were mounted and images were acquired using a Zeiss LSM700 confocal microscope (ZIESS, Germany).

### IF

Cells were seeded on glass coverslips and grown to 60–80% confluence. The cells were washed with 1 × PBS (137 × 10^−3^ m NaCl, 2.7 × 10^−3^ m KCl, 10 × 10^−3^ m Na_2_HPO_4_, 2 × 10^−3^ m KH_2_PO_4_, pH 7.4), fixed with 4% paraformaldehyde, and permeabilized with 0.3% Triton‐X 100 in 1 × PBS. The cells were then blocked with 5% BSA in 1 × PBS. Antibodies were diluted in 1% BSA and 0.3% Triton‐X 100 in 1 × PBS. For immunofluorescence, cells were incubated with rabbit anti‐CAPRIN1(Cell Signaling Technology, USA) and mouse anti‐G3BP1(Cell Signaling Technology, USA) primary antibodies overnight at 4 °C or 2 h at room temperature, followed by three washes with 1 × PBS. The cells were then incubated with Alexa Fluor 594 goat antirabbit (Cell Signaling Technology, USA) and Alexa Fluor 555 goat antimouse (Cell Signaling Technology, USA) secondary antibodies for 2 h at room temperature, followed by three washes with 1 × PBS. The nucleus was then stained by DAPI. The cell slices were mounted and images were acquired using a Zeiss LSM700 confocal microscope (ZIESS, Germany).

### Northern Blot

Northern blot was performed according to a previous study.^[^ ^67^ ^]^ In brief, RNA was isolated from cells using TRIzol reagent (Thermo Fisher Scientific, USA). Biotin labeled probes targeting circVAMP3 and 5.8S rRNA were in vitro transcribed using the HiScribe T7 Quick High Yield RNA Synthesis Kit (NEB, USA) and biotin RNA labeling mix (Roche, Switzerland) according to the manufacturer's protocols. RNA samples were separated by electrophoresis with 8% denaturing urea polyacrylamide gel and then transferred to Hybond‐N+ nylonmembranes (GE Healthcare, USA). The membrane was then incubated with probes overnight. RNA signal was detected using Chemiluminescent Nucleic Acid Detection Module (Thermo Fisher Scientific). The sequences of probes are listed in Table S4 in the Supporting Information.

### RNA Overexpression and Knockdown Assay

For the circVAMP3 overexpression assay, the genomic region of exon 2 of circVAMP3 with its upper 541nt flanking intron and exon 3 of circVAMP3 with its lower 1076 nt flanking intron were amplified by PCR from the DNA of HEK293T cells. Then, a complete fragment was acquired by overlap extension PCR and cloned into the BamHI and NotI sites of the pCDNA3.1+ vector. Huh7 and SMMC‐7721 cells were transfected with recombinant plasmid, cultured for 48 h, and then selected with G418 (300 µg mL^−1^).

For the circVAMP3 silencing assay, sense and antisense DNA oligonucleotides containing shRNA targeting the back‐splice region of circVAMP3 RNA were synthesized, annealed, and inserted into the BamHI and EcoRI sites of the pSIHI‐H1‐puro vector (System Biosciences, Mountain View, CA, USA). To produce lentivirus‐expressing shRNAs, HEK293T cells were cotransfected with pCMV‐VSV‐G, pCMV‐dR8.2 dvpr vectors, and the recombinant vector described above. Forty‐eight hours after 48 h since transfection, supernatants containing lentivirus were harvested and filtered through 0.45 µm filters. SMMC‐7721 and Huh7 cells were infected with lentivirus for 48 h and selected with puromycin (1.5 µg mL^−1^).

### Cell Proliferation, Colony Formation, and Migration Assays

For the cell proliferation assay, cells were seeded into 96‐well flat‐bottomed plates at a density of 2500 cells per well. After 12 h of culture, cell viability was assessed using the CellTiter 96 Aqueous One Solution Cell Proliferation Assay (Promega, USA). EdU immunofluorescence staining was performed using Cell‐Light EdU Apollo567 In Virto Kit (RibioBio, China) according to the manufacturer's instructions. For the colony formation assay, cells were seeded in six‐well plates at a density of 350 cells per well and cultured in complete growth medium at 37 °C for 10 d, fixed with 4% paraformaldehyde, stained with 0.5% crystal violet, and counted.

Migration assays were performed in transwell chambers with 8 µm polycarbonate membranes. Cells in serum‐free medium were seeded into the upper chambers at a density of 5 × 10^4^ cells per chamber and complete medium was added to the lower chambers. After culturing for 24 h, cells that migrated through the membrane were fixed with 4% paraformaldehyde, stained with 0.5% crystal violet, and counted.

### In Vivo Tumor Formation Assay

For the in vivo tumor formation assay, female athymic BALB/c nude mice aged 4–5 weeks were used. 5 × 10^6^ control and circVAMP3 silenced or overexpressed SMMC‐7721 cells were suspended in 200 *μ*L PBS and subcutaneously injected into the right axilla of each nude mouse (*n* = 6 per group). Tumor sizes were measured every 3 d when a tumor was measurable and the volumes were calculated as length × width^2^ × 0.5. After 18 d, the mice were euthanized and tumors were weighed. All experiments were approved by the Animal Care and Use Committee of the Institute of Zoology, Chinese Academy of Sciences.

### RNA Pull‐Down Assay

RNA pull‐down assays were performed as described in the previous study.^[^ ^58^ ^]^ In brief, 10^7^ Huh7 or SMMC‐7721 cells were lysed by cell lysis buffer for western and IP (Beyotime, China) and the supernatants of whole‐cell lysates were incubated with 3 µg 5’‐biotin‐labeled DNA oligo probes against the back‐splice region of circVAMP3 RNA for 1 h at room temperature with gentle rotation. The protein‐RNA complex was captured by incubating with streptavidin C1 magnetic beads (Invitrogen, USA) for 1 h at room temperature with gentle rotation. Then, the complex was washed three times with the lysis buffer and once with wash buffer (5 × 10^−3^ m Tris‐HCl, 0.5× 10^−3^ m EDTA, 1 M NaCl). The RNA in the pull‐down supernatant was isolated by TRIzol and analyzed by RT‐qPCR, and the RNA binding proteins in the pull‐down supernatant were isolated by boiling the beads in 0.1% SDS, and were analyzed by western blotting or mass spectrometry.

### Western Blot

Protein supernatants from cell lysates, pull‐down supernatants, or coimmunoprecipitation supernatants were prepared in 1 × sodium dodecyl sulfate protein loading buffer. Identical quantities of proteins were separated by SDS‐PAGE and transferred onto polyvinylidene fluoride membranes (Millipore, USA). The membranes were blocked with 5% nonfat dried milk in TBST buffer (10 × 10^−3^ m Tris‐HCl, 150 × 10^−3^ m NaCl, 0.1% Tween‐20, pH 7.6) and incubated with protein‐specific primary antibodies. The membranes were then washed three times with TBST buffer and incubated with horseradish peroxidase (HRP)‐conjugated secondary antibodies. The protein signals were visualized using enhanced chemiluminescence reagents (EASYBIO, China), and densitometry values were analyzed using ImageJ software.

### RNA Immunoprecipitation (RIP)

A total of 10^7^ cells were lysed with cell lysis buffer for western blotting and IP (Beyotime, China) containing protease inhibitor cocktail (Roche, Switzerland) and RNA inhibitor (Takara, Japan). The supernatants were collected after centrifugation for 10 min at 13 000 rpm. To capture the protein‐RNA complex, the supernatants were incubated with protein‐specific antibodies overnight at 4 °C with gentle rotation. The complex was incubated with 25 *μ*L protein A/G magnetic beads (Thermo Fisher Scientific, USA) for 2 h at 4 °C with gentle rotation. The beads were washed three times with PBS containing RNase inhibitor and protease inhibitor, and then washed once with nuclease‐and protease‐free water. After washing, the protein‐RNA complexes were eluted, and RNA was extracted using TRIzol reagent (Thermo Fisher Scientific, USA).

### In Vitro Transcription and Cyclization of circVAMP3

The DNA for circVAMP3 synthesis was acquired by PCR using a T7 promoter sequence fused primers. RNA was synthesized in vitro using the HiScribeT7 Quick High Yield RNA Synthesis Kit (NEB, USA) according to the manufacturer's protocols. RNA products were purified using an Agencourt RNAClean XP Kit (Beckman, USA). RNA cyclization was performed as described in a previous study.^[^ ^68^ ^]^ In brief, RNA was mixed with DNA splints at a molar ratio of 1:1.5, incubated at 90 °C for 2 min, and slowly cooled to room temperature. DNA splints act as scaffolds to keep the 5’ and 3’ ends of RNA closer. Then samples were incubated with T4 DNA ligase (NEB, USA) overnight at 16 °C, DNA splint and linear RNA were digested with DNase I (NEB, USA) and RNase R (epicentral, USA), respectively. CircRNAs were purified using an Agencourt RNAClean XP Kit (Beckman, USA). The DNA splint and primer sequences are listed in Table S2 in the Supporting Information.

### FRAP

FRAP experiments were performed using a Zeiss LSM700 confocal microscope (ZIESS, Germany) with a 63 × oil objective. The wtGFP‐CAPRIN1 and mCherry‐G3BP1 genes were synthesized and inserted into the BamHI and EcoRI sites of the pCDNA3.1+ vector. Fluorescent labeled protein condenses were formed in SMMC‐7721 cells by expressing wtGFP‐CAPRIN1 and mCherry‐G3BP1 fused proteins. A circular region with a diameter of 0.8 µm in the center of condensate was bleached to 40% density value using a laser intensity of 80% at 405 nm (for wtGFP‐CAPRIN1) or 560 nm (for mCherry‐G3BP1). Fluorescence intensity of bleaching sites was recorded at 25–35 time points after bleaching (65–80 s). Analysis of the recovery curves were carried out using the ZEN2010 software.

### Protein Expression and Purification

Full‐length 6 × His‐CAPRIN1 and 6 × His‐G3BP1 genes were synthesized and inserted into the NdeI and HindIII sites of the pET30a+ vector (Novagen, Germany). The constructs were transformed into *Escherichia coli* BL21 (DE3) cells. Single colonies were cultured in LB medium containing 50 µg mL^−1^ kanamycin at 37 °C. Expression of histidine tag fused proteins was induced with IPTG (0.5 × 10^−3^ m) at 16 °C for 16 h when the OD_600nm_ of cells reached 0.8. Cells were harvested by centrifugation at 12 000 rpm for 10 min at 4 °C. The cells were then homogenized in resuspension buffer containing 20 × 10^−3^ m Tris‐HCl (pH 8.0), 300 × 10^−3^ m NaCl, 20 × 10^−3^ m imidazole, 1 × 10^−3^ m DTT, and 1 × 10^−3^ m PMSF using a low‐temperature ultrahigh pressure cell disrupter (JNBIO, China). The supernatants were collected by centrifugation at 12 000 rpm for 30 min at 4 °C and filtered through 0.22 µm filters. Thereafter, the supernatants were loaded onto a HisTrap HP column (GE Healthcare, USA). Proteins were gradiently eluted with elution buffer containing 20 × 10^−3^ m Tris‐HCl pH 8.0, 300 × 10^−3^ m NaCl plus 20 × 10^−3^, 50 × 10^−3^, 100 × 10^−3^, and 300 × 10^−3^ m imidazole, respectively, using an AKTA start system (GE Healthcare, USA). Fractions containing CAPRIN1 and G3BP1 were analyzed by SDS‐PAGE and then collected. The buffer of CAPRIN1 and G3BP1 was changed to 25 × 10^−3^ m Tris‐HCl pH 7.3, 150 × 10^−3^ m NaCl by dialysis. Proteins were concentrated and further purified using Superdex 200 PG (GE Healthcare, USA) with a buffer containing 25 × 10^−3^ m Tris‐HCl (pH 7.3) and 150 × 10^−3^ m NaCl. Fractions were analyzed by SDS‐PAGE, concentrated, and stored at ‐80 °C.

### In Vitro LLPS

For general LLPS experiments, purified CAPRIN1 and G3BP1 proteins were incubated at room temperature in LLPS buffer containing 25 × 10^−3^ m Tris‐HCl pH7.3 and 150 × 10^−3^ m NaCl. 3 *μ*L of each sample was transferred onto glass bottom cell culture dishes (NEST, China) and imaged using an Olympus IX83 microscope. For LLPS treated with different concentrations of NaCl, proteins were incubated with 25 × 10^−3^ m Tris‐HCl (pH7.3) and NaCl (50 × 10^−3^–400 × 10^−3^ m). To investigate the influence of different types of RNA on LLPS, tRNA (ThermoFisher, USA), total RNA (isolated and purified from SMMC‐7721 cells), and circVAMP3 RNAs (in vitro transcribed and cyclized) were added to the LLPS samples at a final concentration of 50 ng *μ*L^−1^. Furthermore, 1 *μ*L RNase R (epicentral, USA) or RNase A (Qiagen, Germany) were added to the circVAMP3 RNA‐containing LLPS samples and incubated at room temperature for 10 min to verify the effect of circVAMP3 on LLPS.

### Statistical Analysis

GraphPad Prism 6 and Microsoft Excel software were used for statistical analysis. All experiments that undergo error analysis were carried out in at least three independent replicates. Statistical methods for each result were shown in the corresponding figure legends. Data were presented as mean ± standard deviation except were stated otherwise. * *p* < 0.05, ** *p* < 0.01, or *** *p* < 0.001 were considered as statistically significant.

## Conflict of Interest

The authors declare no conflict of interest.

## Author Contribution

S.C., X.C., and J.Z. contributed equally to this work. F.Z. conceived the project. S.C. performed most of the experiments. X.C. performed the animal experiments and functional experiments in HeLa and A549 cells. J.Z., W.W., and B.Z. carried out the bioinformatics analysis. S.C. and F.Z. wrote the paper.

## Supporting information

Supporting Information

## Data Availability

The data that support the findings of this study are available from the corresponding author upon reasonable request.

## References

[1] J. U. Guo , V. Agarwal , H. Guo , D. P. Bartel , Genome Biol. 2014, 15, 409. 25070500 10.1186/s13059-014-0409-z PMC4165365

[2] L. L. Chen , Nat. Rev. Mol. Cell Biol. 2016, 17, 205. 26908011 10.1038/nrm.2015.32

[3] J. W. Fischer , A. K. Leung , Crit. Rev. Biochem. Mol. Biol. 2017, 52, 220. 28095716 10.1080/10409238.2016.1276882 PMC5526226

[4] J. Salzman , R. E. Chen , M. N. Olsen , P. L. Wang , P. O. Brown , PLoS Genet. 2013, 9, e1003777. 24039610 10.1371/journal.pgen.1003777 PMC3764148

[5] H. Suzuki , Y. Zuo , J. Wang , M. Q. Zhang , A. Malhotra , A. Mayeda , Nucleic Acids Res. 2006, 34, e63. 16682442 10.1093/nar/gkl151 PMC1458517

[6] S. Qu , Y. Zhong , R. Shang , X. Zhang , W. Song , J. Kjems , H. Li , RNA Biol. 2017, 14, 992. 27617908 10.1080/15476286.2016.1220473 PMC5680710

[7] D. Yang , K. Yang , M. Yang , Adv. Exp. Med. Biol. 2018, 1086, 17. 30232750 10.1007/978-981-13-1117-8_2

[8] X. Chen , T. Yang , W. Wang , W. Xi , T. Zhang , Q. Li , A. Yang , T. Wang , Theranostics 2019, 9, 588. 30809295 10.7150/thno.29678 PMC6376182

[9] L. Szabo , R. Morey , N. J. Palpant , P. L. Wang , N. Afari , C. Jiang , M. M. Parast , C. E. Murry , L. C. Laurent , J. Salzman , Genome Biol. 2015, 16, 126. 26076956 10.1186/s13059-015-0690-5 PMC4506483

[10] B. Han , J. Chao , H. Yao , Pharmacol. Ther. 2018, 187, 31. 29406246 10.1016/j.pharmthera.2018.01.010

[11] A. C. Prats , F. David , L. H. Diallo , E. Roussel , F. Tatin , B. Garmy‐Susini , E. Lacazette , Int. J. Mol. Sci. 2020, 21, 8591. 10.3390/ijms21228591 PMC7697609 33202605

[12] H. Sung , J. Ferlay , R. L. Siegel , M. Laversanne , I. Soerjomataram , A. Jemal , F. Bray , CA Cancer J. Clin. 2021, 71, 209. 33538338 10.3322/caac.21660

[13] P. Tabrizian , G. Jibara , B. Shrager , M. Schwartz , S. Roayaie , Ann. Surg. 2015, 261, 947. 25010665 10.1097/SLA.0000000000000710

[14] K. Mao , J. Wang , Zhonghua Wai Ke Za Zhi 2019, 57, 466. 31142072 10.3760/cma.j.issn.0529-5815.2019.06.015

[15] L. S. Kristensen , T. B. Hansen , M. T. Veno , J. Kjems , Oncogene 2018, 37, 555. 28991235 10.1038/onc.2017.361 PMC5799710

[16] X. Sun , X. Ge , Z. Xu , D. Chen , J. Gastroenterol. Hepatol. 2020, 35, 157. 31222831 10.1111/jgh.14762

[17] D. Han , J. Li , H. Wang , X. Su , J. Hou , Y. Gu , C. Qian , Y. Lin , X. Liu , M. Huang , N. Li , W. Zhou , Y. Yu , X. Cao , Hepatology 2017, 66, 1151. 28520103 10.1002/hep.29270

[18] W. W. Du , W. Yang , E. Liu , Z. Yang , P. Dhaliwal , B. B. Yang , Nucleic Acids Res. 2016, 44, 2846. 26861625 10.1093/nar/gkw027 PMC4824104

[19] Z. Li , C. Huang , C. Bao , L. Chen , M. Lin , X. Wang , G. Zhong , B. Yu , W. Hu , L. Dai , P. Zhu , Z. Chang , Q. Wu , Y. Zhao , Y. Jia , P. Xu , H. Liu , G. Shan , Nat. Struct. Mol. Biol. 2015, 22, 256. 25664725 10.1038/nsmb.2959

[20] D. Szklarczyk , A. L. Gable , D. Lyon , A. Junge , S. Wyder , J. Huerta‐Cepas , M. Simonovic , N. T. Doncheva , J. H. Morris , P. Bork , L. J. Jensen , C. V. Mering , Nucleic Acids Res. 2019, 47, D607. 30476243 10.1093/nar/gky1131 PMC6323986

[21] M. V. Kuleshov , M. R. Jones , A. D. Rouillard , N. F. Fernandez , Q. Duan , Z. Wang , S. Koplev , S. L. Jenkins , K. M. Jagodnik , A. Lachmann , M. G. McDermott , C. D. Monteiro , G. W. Gundersen , A. Ma'ayan , Nucleic Acids Res. 2016, 44, W90. 27141961 10.1093/nar/gkw377 PMC4987924

[22] Z. Xie , A. Bailey , M. V. Kuleshov , D. J. B. Clarke , J. E. Evangelista , S. L. Jenkins , A. Lachmann , M. L. Wojciechowicz , E. Kropiwnicki , K. M. Jagodnik , M. Jeon , A. Ma'ayan , Curr. Protoc. 2021, 1, e90. 33780170 10.1002/cpz1.90 PMC8152575

[23] P. Yang , C. Mathieu , R.‐M. Kolaitis , P. Zhang , J. Messing , U. Yurtsever , Z. Yang , J. Wu , Y. Li , Q. Pan , J. Yu , E. W. Martin , T. Mittag , H. J. Kim , J. P. Taylor , Cell 2020, 181, 325. 32302571 10.1016/j.cell.2020.03.046 PMC7448383

[24] H. Matsuki , M. Takahashi , M. Higuchi , G. N. Makokha , M. Oie , M. Fujii , Genes Cells 2013, 18, 135. 23279204 10.1111/gtc.12023

[25] N. Shiina , K. Shinkura , M. Tokunaga , J. Neurosci. 2005, 25, 4420. 15858068 10.1523/JNEUROSCI.0382-05.2005 PMC6725113

[26] J. Y. Youn , W. H. Dunham , S. J. Hong , J. D. R. Knight , M. Bashkurov , G. I. Chen , H. Bagci , B. Rathod , G. MacLeod , S. W. M. Eng , S. Angers , Q. Morris , M. Fabian , J. F. Cote , A. C. Gingras , Mol. Cell 2018, 69, 517. 29395067 10.1016/j.molcel.2017.12.020

[27] N. Kedersha , M. D. Panas , C. A. Achorn , S. Lyons , S. Tisdale , T. Hickman , M. Thomas , J. Lieberman , G. M. McInerney , P. Ivanov , P. Anderson , J. Cell Biol. 2016, 212, 845. 27022092 10.1083/jcb.201508028 PMC4810302

[28] T. H. Kim , B. Tsang , R. M. Vernon , N. Sonenberg , L. E. Kay , J. D. Forman‐Kay , Science 2019, 365, 825. 31439799 10.1126/science.aax4240

[29] R. Wang , L. Cao , R. F. Thorne , X. D. Zhang , J. Li , F. Shao , L. Zhang , M. Wu , Sci. Adv. 2021, 7, eabe5708. 33762340 10.1126/sciadv.abe5708 PMC7990344

[30] P. Anderson , N. Kedersha , Nat. Rev. Mol. Cell Biol. 2009, 10, 430. 19461665 10.1038/nrm2694

[31] W. Wu , P. Ji , F. Zhao , Genome Biol. 2020, 21, 101. 32345360 10.1186/s13059-020-02018-y PMC7187532

[32] J. N. Vo , M. Cieslik , Y. Zhang , S. Shukla , L. Xiao , Y. Zhang , Y. M. Wu , S. M. Dhanasekaran , C. G. Engelke , X. Cao , D. R. Robinson , A. I. Nesvizhskii , A. M. Chinnaiyan , Cell 2019, 176, 869. 30735636 10.1016/j.cell.2018.12.021 PMC6601354

[33] M. A. Altesha , T. Ni , A. Khan , K. Liu , X. Zheng , J. Cell. Physiol. 2019, 234, 5588. 30341894 10.1002/jcp.27384

[34] B. Chen , S. Huang , Cancer Lett. 2018, 418, 41. 29330104 10.1016/j.canlet.2018.01.011

[35] I. L. Patop , S. Kadener , Curr. Opin. Genet. Dev. 2018, 48, 121. 29245064 10.1016/j.gde.2017.11.007 PMC5877416

[36] Q. Zheng , C. Bao , W. Guo , S. Li , J. Chen , B. Chen , Y. Luo , D. Lyu , Y. Li , G. Shi , L. Liang , J. Gu , X. He , S. Huang , Nat. Commun. 2016, 7, 11215. 27050392 10.1038/ncomms11215 PMC4823868

[37] T. B. Hansen , J. Kjems , C. K. Damgaard , Cancer Res. 2013, 73, 5609. 24014594 10.1158/0008-5472.CAN-13-1568

[38] K. Y. Hsiao , Y. C. Lin , S. K. Gupta , N. Chang , L. Yen , H. S. Sun , S. J. Tsai , Cancer Res. 2017, 77, 2339. 28249903 10.1158/0008-5472.CAN-16-1883 PMC5910173

[39] K. Abdelmohsen , A. C. Panda , R. Munk , I. Grammatikakis , D. B. Dudekula , S. De , J. Kim , J. H. Noh , K. M. Kim , J. L. Martindale , M. Gorospe , RNA Biol. 2017, 14, 361. 28080204 10.1080/15476286.2017.1279788 PMC5367248

[40] N. Chen , G. Zhao , X. Yan , Z. Lv , H. Yin , S. Zhang , W. Song , X. Li , L. Li , Z. Du , L. Jia , L. Zhou , W. Li , A. R. Hoffman , J. F. Hu , J. Cui , Genome Biol. 2018, 19, 218. 30537986 10.1186/s13059-018-1594-y PMC6290540

[41] W.‐C. Liang , C.‐W. Wong , P.‐P. Liang , M. Shi , Y. Cao , S.‐T. Rao , S. K.‐W. Tsui , M. M.‐Y. Waye , Q. Zhang , W.‐M. Fu , J.‐F. Zhang , Genome Biol. 2019, 20, 84. 31027518 10.1186/s13059-019-1685-4 PMC6486691

[42] Y. Yang , X. Gao , M. Zhang , S. Yan , C. Sun , F. Xiao , N. Huang , X. Yang , K. Zhao , H. Zhou , S. Huang , B. Xie , N. Zhang , J. Natl. Cancer Inst. 2018, 110, 304. 10.1093/jnci/djx166 PMC6019044 28903484

[43] M. Zhang , K. Zhao , X. Xu , Y. Yang , S. Yan , P. Wei , H. Liu , J. Xu , F. Xiao , H. Zhou , X. Yang , N. Huang , J. Liu , K. He , K. Xie , G. Zhang , S. Huang , N. Zhang , Nat. Commun. 2018, 9, 4475. 30367041 10.1038/s41467-018-06862-2 PMC6203777

[44] M. Zhang , N. Huang , X. Yang , J. Luo , S. Yan , F. Xiao , W. Chen , X. Gao , K. Zhao , H. Zhou , Z. Li , L. Ming , B. Xie , N. Zhang , Oncogene 2018, 37, 1805. 29343848 10.1038/s41388-017-0019-9

[45] P. Li , S. Banjade , H. C. Cheng , S. Kim , B. Chen , L. Guo , M. Llaguno , J. V. Hollingsworth , D. S. King , S. F. Banani , P. S. Russo , Q. X. Jiang , B. T. Nixon , M. K. Rosen , Nature 2012, 483, 336. 22398450 10.1038/nature10879 PMC3343696

[46] Y. Shin , C. P. Brangwynne , Science 2017, 357, eaaf4382. 28935776 10.1126/science.aaf4382

[47] V. N. Uversky , Curr. Opin. Struct. Biol. 2017, 44, 18. 27838525 10.1016/j.sbi.2016.10.015

[48] A. H. Fox , S. Nakagawa , T. Hirose , C. S. Bond , Trends Biochem. Sci. 2018, 43, 124. 29289458 10.1016/j.tibs.2017.12.001

[49] Y. T. Sasaki , T. Ideue , M. Sano , T. Mituyama , T. Hirose , Proc. Natl. Acad. Sci. U. S. A. 2009, 106, 2525. 19188602 10.1073/pnas.0807899106 PMC2650297

[50] S. Souquere , G. Beauclair , F. Harper , A. Fox , G. Pierron , Mol. Biol. Cell 2010, 21, 4020. 20881053 10.1091/mbc.E10-08-0690 PMC2982136

[51] Y. Gao , G. Pei , D. Li , R. Li , Y. Shao , Q. C. Zhang , P. Li , Cell Res. 2019, 29, 767. 31388144 10.1038/s41422-019-0210-3 PMC6796879

[52] R. J. Ries , S. Zaccara , P. Klein , A. Olarerin‐George , S. Namkoong , B. F. Pickering , D. P. Patil , H. Kwak , J. H. Lee , S. R. Jaffrey , Nature 2019, 571, 424. 31292544 10.1038/s41586-019-1374-1 PMC6662915

[53] A. Aulas , M. M. Fay , S. M. Lyons , C. A. Achorn , N. Kedersha , P. Anderson , P. Ivanov , J. Cell Sci. 2017, 130, 927. 28096475 10.1242/jcs.199240 PMC5358336

[54] D. S. W. Protter , R. Parker , Trends Cell Biol. 2016, 26, 668. 27289443 10.1016/j.tcb.2016.05.004 PMC4993645

[55] S. Namkoong , A. Ho , Y. M. Woo , H. Kwak , J. H. Lee , Mol. Cell 2018, 70, 175. 29576526 10.1016/j.molcel.2018.02.025 PMC6359928

[56] C. McCormick , D. A. Khaperskyy , Nat. Rev. Immunol. 2017, 17, 647. 28669985 10.1038/nri.2017.63

[57] S. Solomon , Y. Xu , B. Wang , M. D. David , P. Schubert , D. Kennedy , J. W. Schrader , Mol. Cell. Biol. 2007, 27, 2324. 17210633 10.1128/MCB.02300-06 PMC1820512

[58] P. Ji , W. Wu , S. Chen , Y. Zheng , L. Zhou , J. Zhang , H. Cheng , J. Yan , S. Zhang , P. Yang , F. Zhao , Cell Rep. 2019, 26, 3444. 30893614 10.1016/j.celrep.2019.02.078

[59] Y. Li , Y. Z. Ge , L. Xu , R. Jia , Life Sci. 2020, 254, 117176. 31843532 10.1016/j.lfs.2019.117176

[60] Y. Yang , L. Chen , J. Gu , H. Zhang , J. Yuan , Q. Lian , G. Lv , S. Wang , Y. Wu , Y. T. Yang , D. Wang , Y. Liu , J. Tang , G. Luo , Y. Li , L. Hu , X. Sun , D. Wang , M. Guo , Q. Xi , J. Xi , H. Wang , M. Q. Zhang , Z. J. Lu , Nat. Commun. 2017, 8, 14421. 28194035 10.1038/ncomms14421 PMC5316832

[61] D. Kim , B. Langmead , S. L. Salzberg , Nat. Methods 2015, 12, 357. 25751142 10.1038/nmeth.3317 PMC4655817

[62] M. Pertea , G. M. Pertea , C. M. Antonescu , T. C. Chang , J. T. Mendell , S. L. Salzberg , Nat. Biotechnol. 2015, 33, 290. 25690850 10.1038/nbt.3122 PMC4643835

[63] M. D. Robinson , D. J. McCarthy , G. K. Smyth , Bioinformatics 2010, 26, 139. 19910308 10.1093/bioinformatics/btp616 PMC2796818

[64] H. Li arXiv, 2013. https://arxiv.org/abs/1303.3997

[65] Y. Gao , J. Zhang , F. Zhao , Briefings Bioinf. 2018, 19, 803. 10.1093/bib/bbx014 28334140

[66] J. Zhang , S. Chen , J. Yang , F. Zhao , Nat. Commun. 2020, 11, 90. 31900416 10.1038/s41467-019-13840-9 PMC6941955

[67] C. Li , P. D. Zamore , Cold Spring Harbor Protoc. 2018, 2018. 10.1101/pdb.prot097543 29438062

[68] Q. Li , Y. Wang , S. Wu , Z. Zhou , X. Ding , R. Shi , R. F. Thorne , X. D. Zhang , W. Hu , M. Wu , Cell Metab. 2019, 30, 157. 31155494 10.1016/j.cmet.2019.05.009

