# Supplementary material for: circVAMP3 Drives CAPRIN1 Phase Separation and Inhibits Hepatocellular Carcinoma by Suppressing c‐Myc Translation
Source: Adv Sci (Weinh). 2022 Jan 24;9(8):2103817. doi: 10.1002/advs.202103817 (PMC8922094; doi:10.1002/advs.202103817)
Supplement: Supplementary file 1 — Supporting Information [file ADVS-9-2103817-s001.pdf]

ADVANCED SCIENCE
Open Access

Supporting Information

for *Adv. Sci.*, DOI 10.1002/advs.202103817

circVAMP3 Drives CAPRIN1 Phase Separation and Inhibits Hepatocellular Carcinoma by Suppressing c-Myc Translation

*Shuai Chen, Xiaofei Cao, Jinyang Zhang, Wanying Wu, Bing Zhang and Fangqing Zhao**

Supporting Information

for *Adv. Sci.,* DOI: 10.1002/advs.202103817

circVAMP3 drives CAPRIN1 phase separation and inhibits hepatocellular carcinoma by suppressing c-Myc translation

*Shuai Chen, Xiaofei Cao, Jinyang Zhang, Wanying Wu, Bing Zhang, Fangqing Zhao**

Supporting Information

circVAMP3 drives CAPRIN1 phase separation and inhibits hepatocellular carcinoma by suppressing c-Myc translation

Shuai Chen, Xiaofei Cao, Jinyang Zhang, Wanying Wu, Bing Zhang, Fangqing Zhao[*]

Supplementary Figures and Figure legends

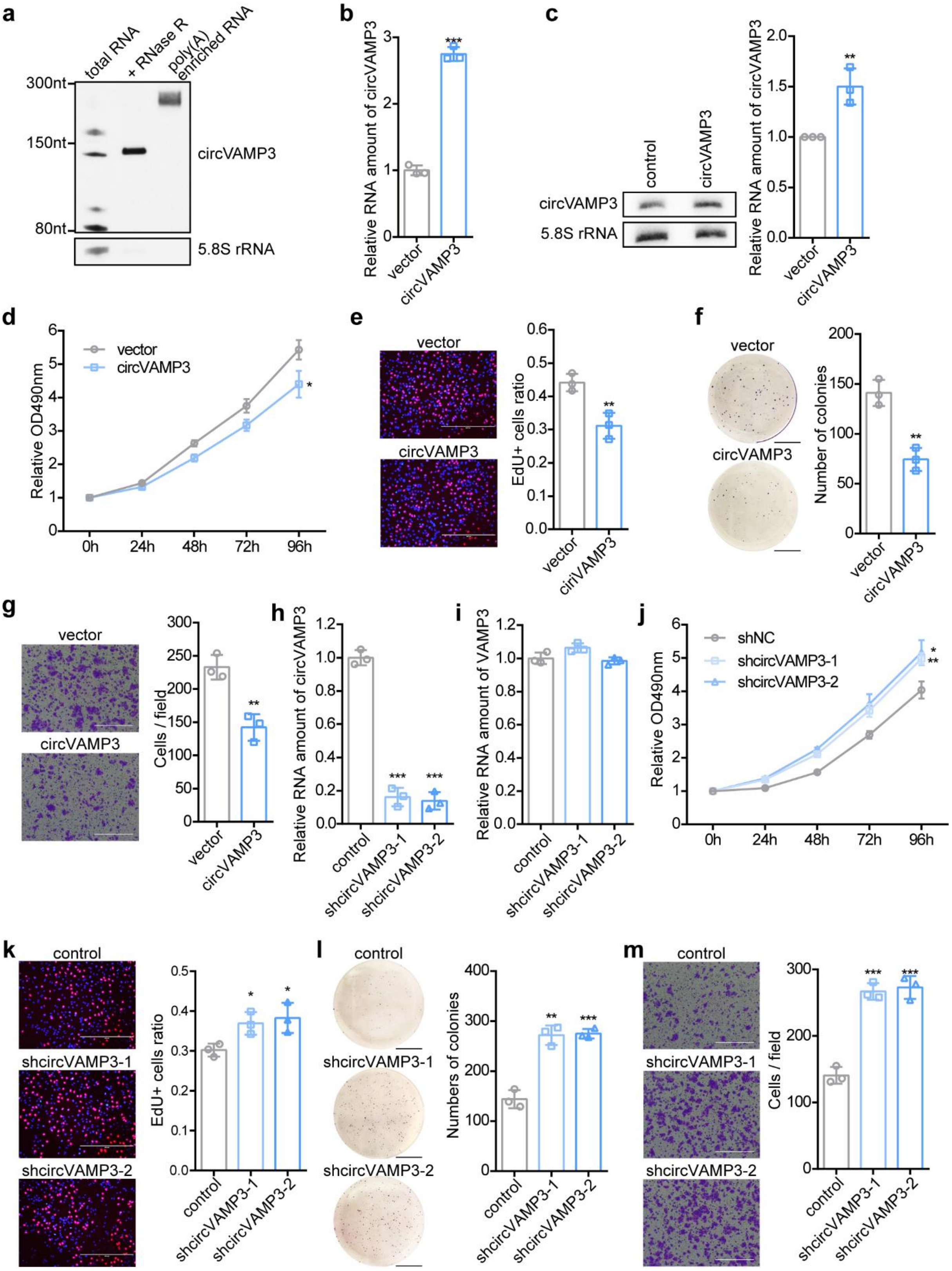

Figure. S1. circVAMP3 inhibits Huh7 cell proliferation, growth, and metastasis. **a**) Northern blot analysis of circVAMP3 in total RNA, RNase R treated RNA and poly(A) enriched RNA.

b) Relative RNA level of circVAMP3 in Huh7 cells stably overexpressing circVAMP3 normalized to GAPDH. **c**) Northern blot analysis revealed the RNA level of circVAMP3 in Huh7 cells stably overexpressing circVAMP3 normalized to 5.8S rRNA. **d, e**) Cell proliferation of Huh7 cells stably overexpressing circVAMP3 using MTS assay **(d)** and EdU staining assay **(e)**. **f**) Colony formation assay of Huh7 cells stably overexpressing circVAMP3. **g)** Cell metastasis of Huh7 cells stably overexpressing circVAMP3 using transwell cell migration assay. **h, i**) Relative RNA level of circVAMP3 **(h)** and VAMP3 **(i)** in Huh7 cells stably silencing circVAMP3 normalized to GAPDH. **j, k**) Cell proliferation of Huh7 cells stably silencing circVAMP3 using MTS assay **(j)** and EdU staining assay **(k)**. **l**) Colony formation assay of Huh7 cells stably silencing circVAMP3. **m**) Cell metastasis of Huh7 cells stably silencing circVAMP3 using transwell cell migration assay. Data in **b - m** are presented as mean ± SD, n = 3. * $P < 0.05$, ** $P < 0.01$, *** $P < 0.001$ by two-tailed unpaired Student t test. Scale bars in **e**, **g**, **k** and **m** are 400 μm; scale bars in **f** and **i** are 1 cm.

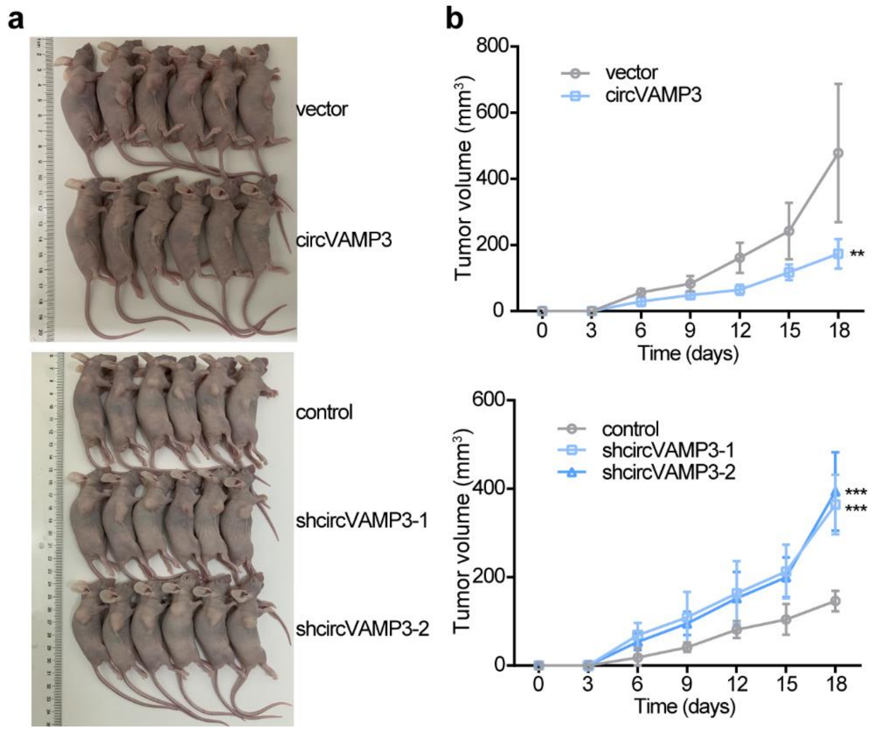

Figure. S2. circVAMP3 inhibits SMMC-7721 cell proliferation in vivo. **a**) The representative images of nude mice that was subcutaneously injected with circVAMP3 overexpressed or silenced and control SMMC-7721 cells. **b**) The tumor growth curve of xenografts in **a**. Data are expressed as mean ± SD, n = 6. ** P < 0.01, *** P < 0.001 by two-tailed unpaired Student t test.

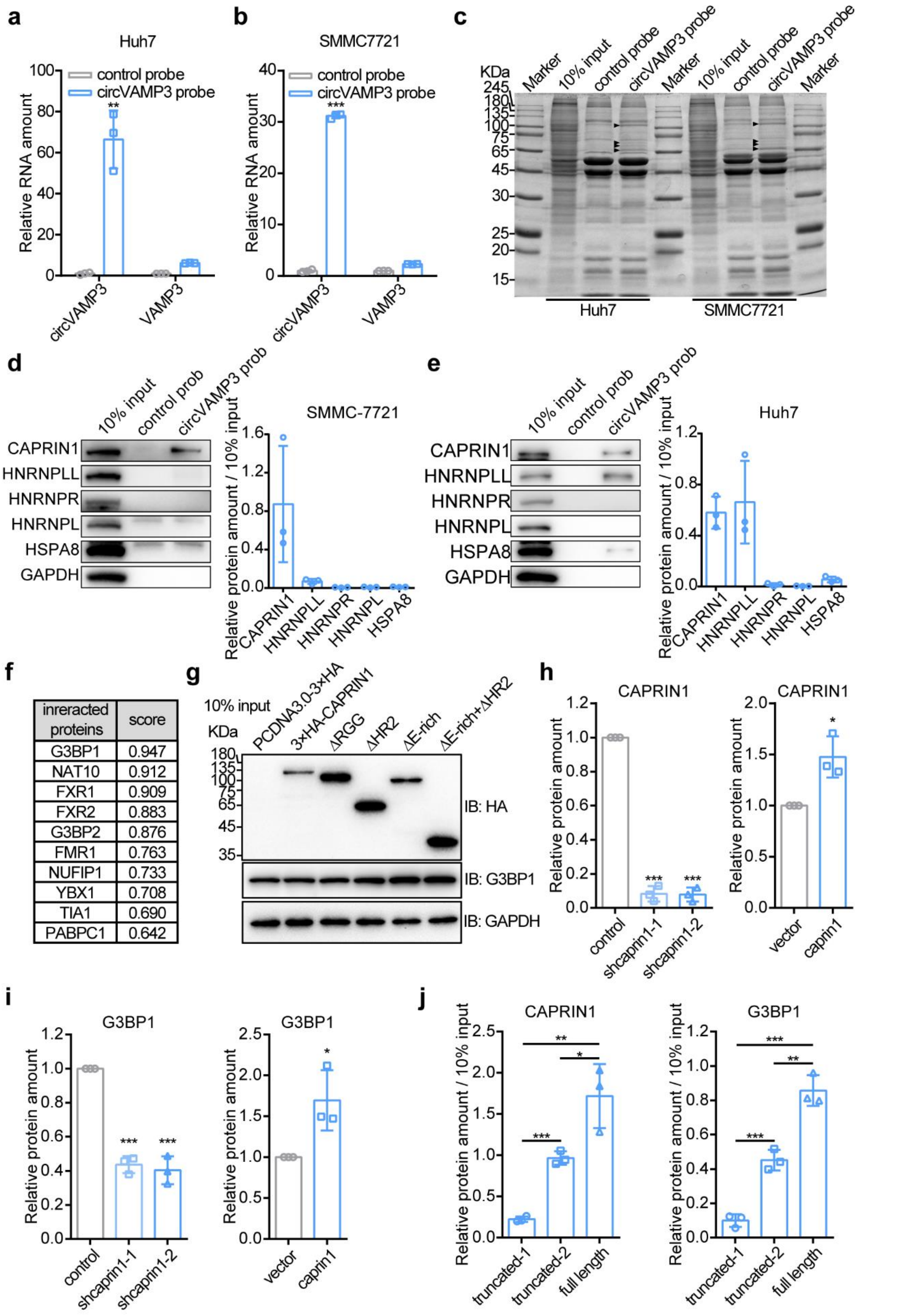

Figure. S3. RNA pull-down results of circVAMP3 and the proteins interacted with circVAMP3. **a, b**) Relative RNA level of circVAMP3 and VAMP3 in the RNA pull-down

products in Huh7 (**a**) and SMMC-7721 (**b**) cells. Control probe, probes that target GFP; circVAMP3 probe, probes that target the back splicing sites of circVAMP3. **c**) SDS-PAGE analysis of circVAMP3 binding proteins in Huh7 and SMMC-7721 cells. The gel was stained by coomassie brilliant blue R250, the specific bands (marked by black triangle) were analyzed by mass spectrometry. **d**, **e**) Immunoblotting analysis of five proteins in RNA pull-down samples by circVAMP3 probes in cytoplasmic lysates of SMMC-7721 (**d**) and Huh7 (**e**) cells. **f**) Proteins that interact with CAPRIN1 and their interacting scores. **g**) Immunoblotting analysis of truncated 3 × HA-tagged CAPRIN1 and G3BP1 in SMMC-7721 cells that express truncated CAPRIN1. GAPDH is served as the endogenous reference control. **h)** Relative protein amount of CAPRIN1 in CAPRIN1 depleted or overexpressed SMMC-7721 cells, which was normalized to GAPDH. **i)** Relative protein amount of G3BP1 in RNA pull-down samples by circVAMP3 probes in CAPRIN1 depleted or overexpressed SMMC-7721 cells normalized to GAPDH. **j)** Relative protein amount of CAPRIN1 and G3BP1 in RNA pull-down samples by biotin-labeled full-length or truncated circVAMP3 in SMMC-7721 cells. Data in **a**, **b**, **d**, **e**, **h-j** are expressed as mean ± SD, n = 3. * $P < 0.05$, ** $P < 0.01$, *** $P < 0.001$ by two-tailed unpaired Student t test.

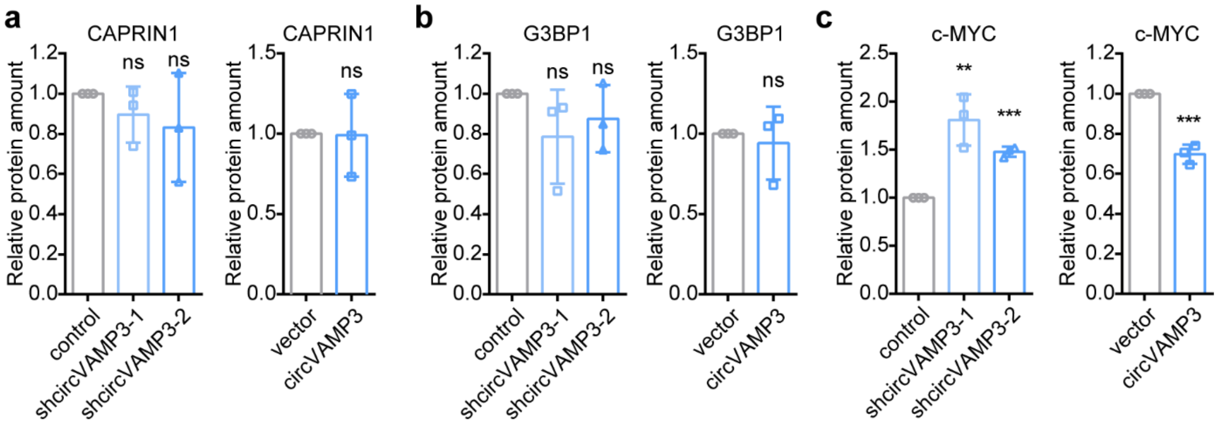

Figure. S4. Relative protein amount of CAPRIN1 **(a)**, G3BP1 **(b)** and c-MYC **(c)** in circVAMP3 depleted or overexpressed SMMC-7721 cells normalized to GAPDH. Data in **a-c** are expressed as mean ± SD, n = 3. ns P > 0.05, * P < 0.05, ** P < 0.01, *** P < 0.001 by two-tailed unpaired Student t test.

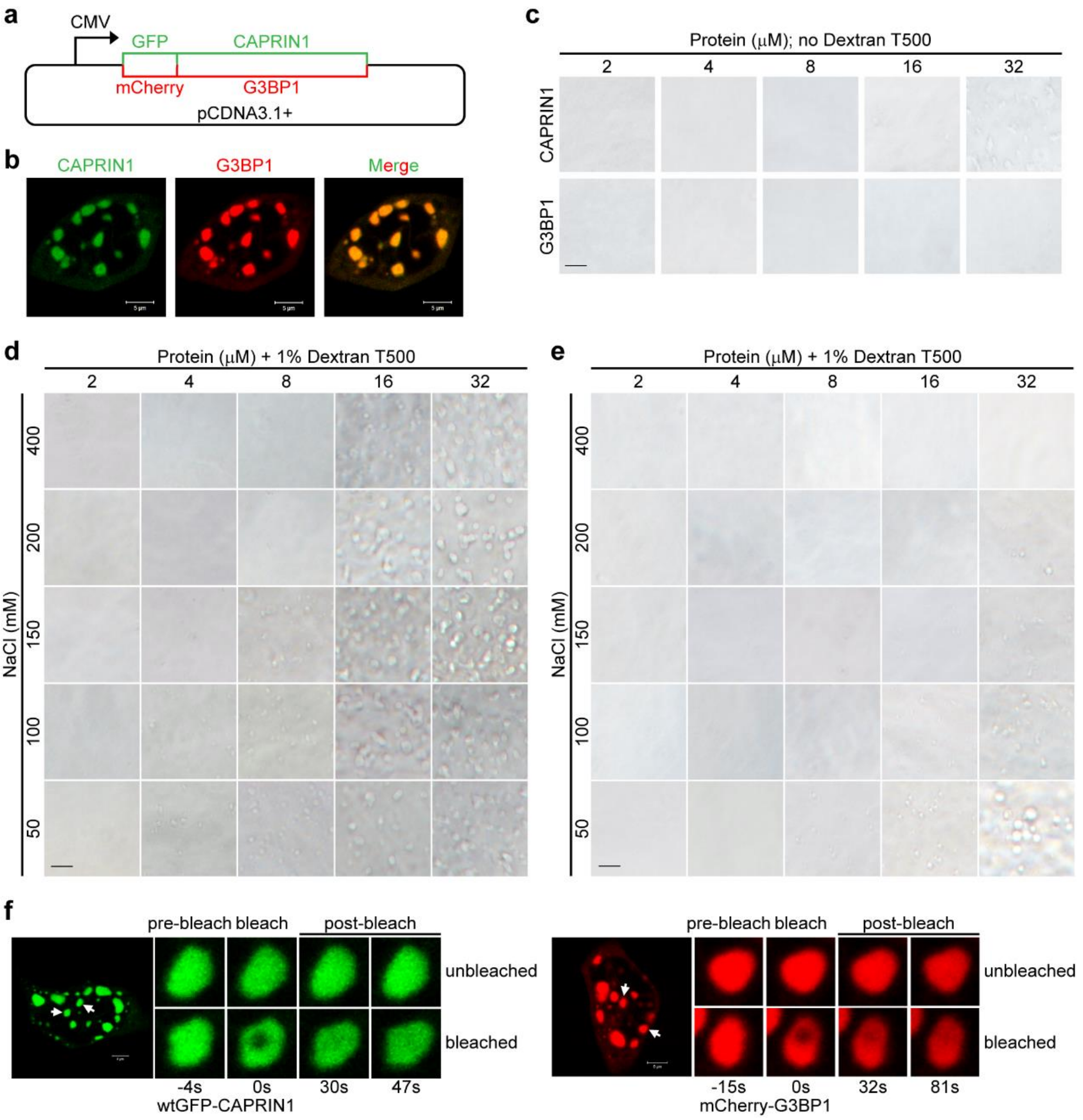

Figure. S5. Liquid-like properties of CAPRIN1 and G3BP1 condensates and droplets. **a**) Ideograph of GFP-CAPRIN1 and mCherry-G3BP1 overexpressing vector. **b**) Subcellular co-localization of GFP-CAPRIN1 and mCherry-G3BP1 expressed in SMMC-7721 cells. **c**) Phase separation of purified CAPRIN1 and G3BP1 in 150 mM NaCl without dextran T500 (crowding agent). **d**, **e**) Phase separation behaviors of different concentrations of purified CAPRIN1 (**d**) and G3BP1 (**e**) in different concentrations of NaCl with the addition of 1% dextran T500 (crowding agent). **f**) Representative images of fluorescence recovery of CAPRIN1 and G3BP1 granules in SMMC-7721 cells. Scale bars in **b** and **f** are 5 μm; in **c** - **e** are 20 μm.

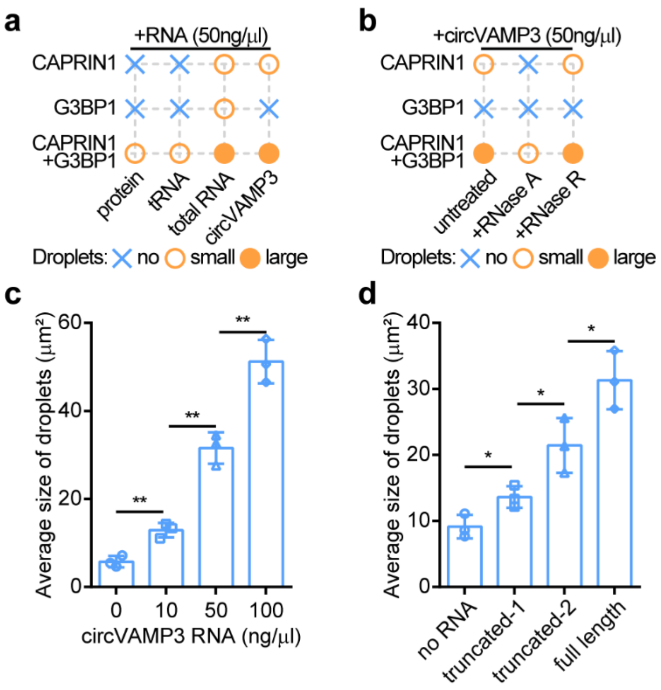

Figure. S6. Quantification of the ability of circVAMP3 in promoting phase separation of CAPRIN1. **a)** Diagrams of phase separation results of CAPRIN1 and G3BP1 under different types of RNAs. **b)** Diagrams of phase separation results of CAPRIN1 and G3BP1 under the condition of circVAMP3 and different RNase. **c)** Average size of CAPRIN1 and G3BP1 protein droplets under different concentration of circVAMP3. **c)** Average size of CAPRIN1 and G3BP1 protein droplets under different truncated circVAMP3. Data in **c** and **d** are expressed as mean ± SD, n = 3. * $P < 0.05$, ** $P < 0.01$ by two-tailed unpaired Student t test.

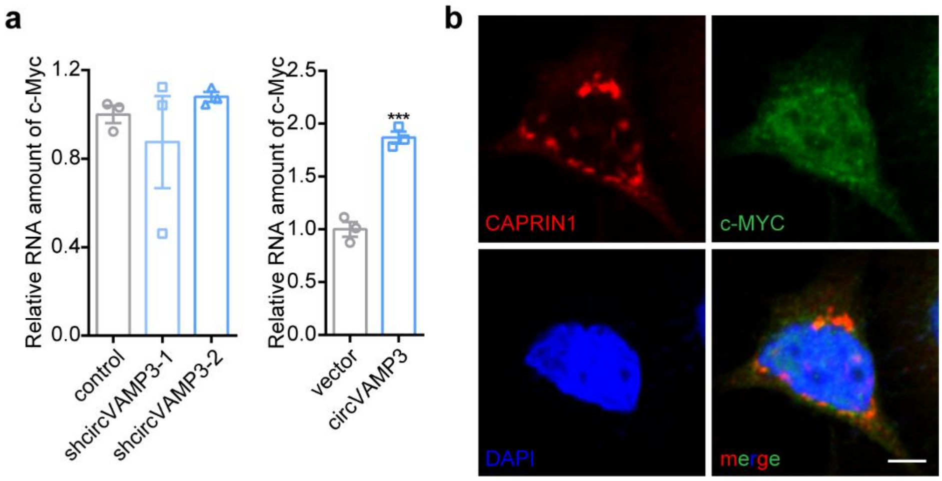

Figure. S7. The effects of circVAMP3 on c-Myc gene expression and sub cellular localization of c-MYC protein. **a)** Relative RNA level of c-Myc in SMMC-7721 cells stably silencing (left) or overexpressing (right) circVAMP3 normalized to GAPDH. Data are expressed as mean ± SEM, n = 3. *** $P < 0.001$ by two-tailed unpaired Student t test. **b)** Immunofluorescence of CAPRIN1 and c-MYC proteins in SMMC-7721 cells treated by sodium arsenite (100 μM, 40 min). Scale bar, 5 μm.

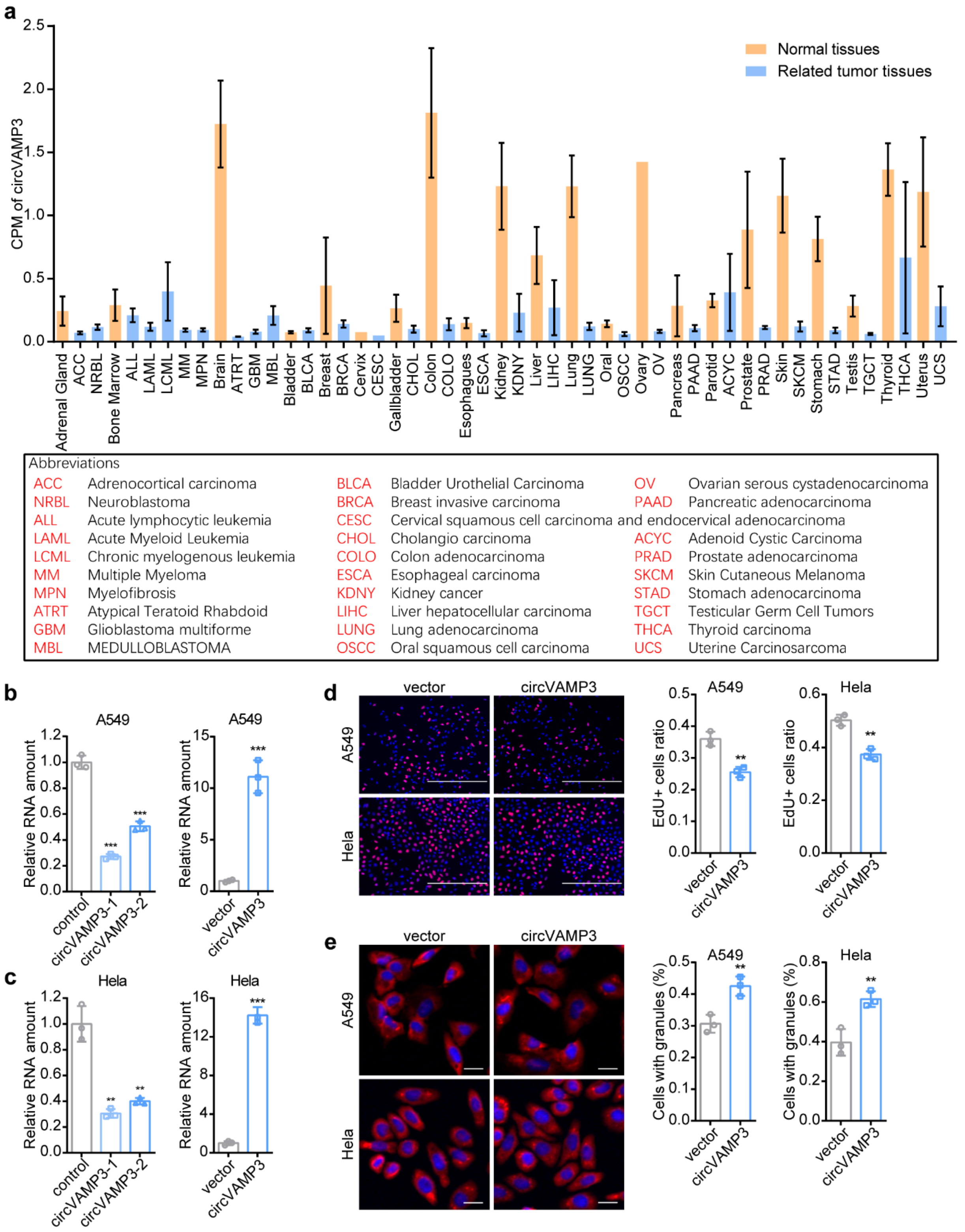

Figure. S8. The reduced expression of circVAMP3 in various cancers and the tumor suppressor function of circVAMP3 in other cancers. **a**) Expression level of circVAMP3 in normal human tissues and their corresponding tumor tissues. Data are expressed as mean ± SEM. **b, c**) Relative RNA level of circVAMP3 in A549 (**b**) and Hela (**c**) cells stably silencing

or overexpressing circVAMP3 normalized to GAPDH. **d**) Cell proliferation of control and circVAMP3 overexpressed A549 and Hela cells using EdU staining assay. Scale bar, 400 μm. **e**) Left: Immunofluorescence of caprin1 in control and circVAMP3 overexpressed A549 and Hela cells treated with sodium arsenite (500 μM for A549 and 100 μM for Hela, 40 min). Scale bar, 20 μm. Right: The percentage of cells with stress granules. Data in **b**-**e** are expressed as mean ± SD, n = 3. ** $P < 0.01$, *** $P < 0.001$ by two-tailed unpaired Student t test.

Table. S1. Sequencing statistics of 20 pairs of HCC and adjacent samples.

Sample	Reads	Mapped Reads	CircRNA Reads	#circRNAs	Sequencing Depth (Exon)	Sequencing Depth (Intron)	Sequencing Depth (Intergenic)
N3	136,694,980	130,450,269	47,632	3,844	81.71	8.29	1.82
N6	139,597,290	132,711,222	50,796	4,021	106.10	7.62	2.14
N7	123,330,708	117,456,674	75,782	5,525	93.28	6.55	1.52
N8	129,194,346	123,716,776	39,090	3,248	98.23	6.52	1.82
N10	139,006,986	133,642,378	46,106	3,640	112.02	7.31	2.00
N11	253,245,058	245,532,735	139,872	8,016	232.83	14.56	4.74
N12	116,407,786	112,504,597	81,498	5,549	118.32	6.47	2.20
N13	187,084,262	182,109,138	113,736	7,308	184.61	10.52	3.57
N14	123,091,034	119,149,401	97,862	6,014	143.31	6.61	3.11
N15	160,014,704	156,146,540	99,476	6,349	149.16	8.22	2.86
N16	296,118,742	286,250,814	307,106	14,195	260.20	16.58	4.30
N17	266,866,632	257,479,273	173,570	10,257	262.06	15.30	4.93
N18	159,813,190	152,602,077	72,276	4,794	153.69	8.73	2.41
N19	152,497,360	146,006,770	103,016	6,704	140.50	7.88	2.43
N20	155,449,102	149,092,879	78,878	5,383	133.75	7.80	2.47
N21	163,608,324	148,931,798	65,060	4,949	130.39	8.34	2.61
N22	162,619,316	155,930,780	66,822	4,828	122.24	8.71	2.60
N24	156,745,684	152,778,858	106,850	6,868	145.08	8.26	2.24
N25	120,960,148	117,444,440	52,328	3,902	120.19	6.56	1.86
N26	167,586,254	163,475,387	98,226	6,508	131.66	9.56	2.68
T3	115,572,400	109,342,368	46,166	3,480	90.13	5.86	1.91
T6	150,683,938	143,358,484	46,902	3,600	127.23	6.83	1.89
T7	142,282,836	134,720,704	67,060	5,342	104.36	7.33	2.04
T8	161,904,068	155,090,513	43,046	3,569	129.21	8.45	2.60
T10	163,245,192	156,988,750	59,362	4,405	97.38	9.05	2.62
T11	101,577,720	98,018,836	50,670	3,728	91.93	5.11	1.31
T12	217,799,148	210,118,754	149,828	8,871	206.86	9.94	3.75
T13	132,541,024	128,862,119	57,404	4,595	108.49	6.43	1.90
T14	187,619,442	180,844,592	175,458	10,759	161.92	9.25	2.61
T15	139,813,652	136,016,317	60,598	4,177	135.14	6.29	2.09
T16	120,636,918	115,786,942	96,756	6,541	118.24	5.41	1.93
T17	178,046,126	171,572,777	88,808	6,320	162.13	10.64	2.74
T18	127,214,848	121,985,986	67,758	4,295	98.57	6.28	2.61
T19	170,747,910	164,632,907	60,170	4,729	155.60	7.99	2.61
T20	175,719,462	169,462,128	49,058	3,654	143.14	8.72	3.04
T21	148,579,482	143,497,368	67,770	5,085	126.25	7.45	2.20
T22	167,387,130	160,971,771	69,050	4,779	139.86	8.41	2.46
T24	166,158,612	159,542,255	110,244	6,394	153.62	7.82	3.37
T25	141,304,054	138,231,817	91,948	6,484	127.88	7.01	2.59

T26	179,716,342	174,440,276	98,116	6,078	153.86	9.52	2.71

Note: N represents normal sample; T represents tumor sample.

Table. S2. Relationships between circVAMP3 expression and clinicopathological characters of HCC patients

**Parameter**	**Numbers of patients**	**circVAMP3(low)**	**circVAMP3(high)**	***p*-value**
**sex**				0.257
male	97	47	50	
female	21	13	8	
**Age (year)**				0.223
<60	77	36	41	
≥60	41	24	17	
**TNM stage**				**0.029**
I+II	81	35	46	
III+IV	37	24	13	
**Tumor size (cm)**				**0.024**
＜5	72	30	42	
≥5	46	29	17	
**Tumor numbers**				0.509
1	108	53	55	
>1	10	6	4	
**AFP (ng/ml)**				0.426
≤400	85	42	43	
>400	33	19	14	
**HBsAg**				0.205
negative	30	18	12	
positive	88	41	47	
**PVTT**				0.355
negative	53	24	29	
positive	65	35	30	
**vessel invasion**				**0.014**
no	20	5	15	
yes	98	54	44	

Abbreviations: AFP, alpha-fetoprotein; PVTT, portal vein tumor thrombus.

Table. S3. Mass Spectrometry results for circVAMP3 RNA pull-down experiments in Huh7 and SMMC-7721 cells.

**Accession**	**Protein**	**Peptides / Unique peptides**	**Molecular weight**	**Score**

		Huh7	SMMC-7721	(KDa)	Huh7	SMMC-7721
Q6NTA2	HNRNPL	22/1	12/11	61.9	128.00	41.80
P11142	Heat shock cognate	11/9	12/10	68.8	37.46	40.39
B7WPG3	Heterogeneous nuclear ribonucleoprotein L-like	9/8	5/4	56.4	18.20	10.41
Q14444	CAPRIN-1	6/6	6/6	78.3	22.56	25.66
Q0VGD6	HNRNPR	4/2	4/2	67.8	13.22	20.72

Table. S4. Primers, probes and shRNAs used in this study.

List of oligonucleotides	Sequences (5’ → 3’)
**Primers for PCR and RT-qPCR**	
VAMP3-convergent-F	TTGGCATTATTTTCAGAACCACA
VAMP3-convergent-R	CAAAAGCTCTTCACCAGCATCTTA
VAMP3-divergent-F	CGACCGTGCAGACGCACT
VAMP3-divergent-R	TCGCATTATGTCCACCACCG
qU1-F	GGGAGATAACGTGACCACGAAG
qU1-R	CCACAAATTATGCAGTCGAGTTTC
qactin-F	CATGTACGTTGCTATCCAGGC
qactin-R	CTCCTTAATGTCACGCACGAT
qGAPDH-F	TGACATCAAGAAGGTGGTGA
qGAPDH-R	TCCACCACCCTGTTGCTGTA
qcircVAMP3-F	CGACCGTGCAGACGCACT
qcircVAMP3-R	TCGCATTATGTCCACCACCG
**Primers for plasmid construction**	
circVAMP3-BamHI-F	atgcttggatccGATATTAGTTGTGGA
circVAMP3-NotI-R	atgcttgcggccgcGCCAGGCACCAAC
circVAMP3-overlap-R	CAGCCAAGTTGAAGAGGAAATATTGGTGGAAGAATTGCA AGATGTGGGCAATCGGG
circVAMP3-overlap-F	ATGATGAAGATAACCAGAACAGTAATCCCGATTGCCCACA TCTTGCAATTCTTCCA
CAPRIN1-BamHI-F	atgcttggatccATGCCCTCGGCCACCAGC
CAPRIN1-EcoRI-R	gcagaattcTTAATTCACTTGCTGAGTGTTC
G3BP1-BamHI-F	atgcttggatccATGGTGATGGAGAAGCCTAG
G3BP1-EcoRI-R	gcagaattcTCACTGCCGTGGCGCAAGC
GFP-pCDNA-F	ttggtaccgagctcggatccGCCACCATGGTGAGCAAGGGCGAGGAG C
GFP-CAPRIN1-R	gtggccgagggcatggatccCTTGTACAGCTCGTCCATGCCG
mCherry-pCDNA-F	ttggtaccgagctcggatccGCCACCATGGTGAGCAAGGGCGAGGAG C
mCherry-G3BP1-R	ttctccatcaccatggatccCTTGTACAGCTCGTCCATGCCG

**Oligos for RNA FISH**	
T7-circVAMP3-prob-F	TAATACGACTCACTATAGGGTTATGTCCACCACCGATGATG T
T7-circVAMP3-prob-R	ACATCATCGGTGGTGGACATAACCCTATAGTGAGTCGTATT A
T7-c-Myc-prob-F	TAATACGACTCACTATAGCGTAGTTGTGCTGATGTG
T7-c-Myc-prob-R	CACATCAGCACAACTACGCTATAGTGAGTCGTATTA
**RNA pull-down probes**	
B-circVAMP3	biotin-aaaATGTCCACCACCGATGATG
B-control	biotin-aaaTGCGCTCCTGGACGTAGCC
**Northern probes**	
circVAMP3-northern	GTAATCCCGATTGCCCACATCTTGCAATTCTTCCACCAAT
5.8S-northern	TCGCAGCTAGCTGCGTTCTTCATCGACGCACGAGCCGAGT GATCCACCGCTAAGAGTCG
**shRNAs**	
shVAMP3_1F	gatccCATCGGTGGTGGACATAATcttcctgtcagaATTATGTCCACC ACCGATGtttttg
shVAMP3_1R	aattcaaaaaCATCGGTGGTGGACATAATtctgacaggaagATTATGTC CACCACCGATGg
shVAMP3_2F	gatccCATCATCGGTGGTGGACATcttcctgtcagaATGTCCACCAC CGATGATGtttttg
shVAMP3_2R	aattcaaaaaCATCATCGGTGGTGGACATtctgacaggaagATGTCCAC CACCGATGATGg
**Primers and oligos for in vitro transcription of full-length and truncated circVAMP3**	
T7-circVAMP3-truncated-1-F	TAATACGACTCACTATAGGGTGTTCTGGTTATCTTCATCAT
circVAMP3-truncated-1-R	TTGTCCACGTTAACTCGCATT
T7-circVAMP3-truncated-2-F	TAATACGACTCACTATAGGGATATTGGTGGAAGAATTGCAA
circVAMP3-truncated-2-R	CGGTCGTCTAACTCAGAGAGC
T7-circVAMP3-full-length-F	TAATACGACTCACTATAGGTGGTGGACATAATGCG
circVAMP3-full-length-R	GATGATGATGATGATGAAG
circVAMP3-full-length-splint	TTAACTCGCATTATGTCCACCACCGATGATGATGATGATGA AGATA

Table. S5. Antibodies used in this study.

Antibody Name	Company	Catalog Number
CAPRIN1	Proteintech	15112-1-AP
G3BP1	Proteintech	13057-2-AP

G3BP1	Proteintech	66486-1-AP
c-MYC	Proteintech	10828-1-AP
HA-tag	EASYBIO	BE2008
GAPDH	EASYBIO	BE0023
β-ACTIN	EASYBIO	BE0021
Anti-Mouse IgG-HRP conjugated	EASYBIO	BE0102
Anti-Rabbit IgG-HRP conjugated	EASYBIO	BE0101
His-tag mAb-HRP-DirecT	MBL	D291-7
Phosphor-eIF2α (Ser51)	Cell Signaling Technology	＃3398
Anti-Rabbit IgG (Alexa Fluor 488 conjugate)	Cell Signaling Technology	＃4412
Anti-Rabbit IgG (Alexa Fluor 555 conjugate)	Cell Signaling Technology	＃4413
Anti-Mouse IgG (Alexa Fluor 555 conjugate)	Cell Signaling Technology	＃4409
Anti-Rabbit IgG (Alexa Fluor 594 conjugate)	Cell Signaling Technology	＃8889
Anti-Mouse IgG (Alexa Fluor 594 conjugate)	Cell Signaling Technology	＃8890
Anti-Biotin (FITC conjugate)	abcam	ab6650
